# A Booster Dose of CoronaVac Increases Neutralizing Antibodies and T Cells that Recognize Delta and Omicron Variants of Concern

**DOI:** 10.1128/mbio.01423-22

**Published:** 2022-08-10

**Authors:** Bárbara M. Schultz, Felipe Melo-González, Luisa F. Duarte, Nicolás M. S. Gálvez, Gaspar A. Pacheco, Jorge A. Soto, Roslye V. Berríos-Rojas, Liliana A. González, Daniela Moreno-Tapia, Daniela Rivera-Pérez, Mariana Ríos, Yaneisi Vázquez, Guillermo Hoppe-Elsholz, Catalina A. Andrade-Parra, Omar P. Vallejos, Alejandro Piña-Iturbe, Carolina Iturriaga, Marcela Urzua, María S. Navarrete, Álvaro Rojas, Rodrigo Fasce, Jorge Fernández, Judith Mora, Eugenio Ramírez, Aracelly Gaete-Argel, Mónica L. Acevedo, Fernando Valiente-Echeverría, Ricardo Soto-Rifo, Daniela Weiskopf, Alba Grifoni, Alessandro Sette, Gang Zeng, Weining Meng, CoronaVac03CL Study Group, José V. González-Aramundiz, Pablo A. González, Katia Abarca, Alexis M. Kalergis, Susan M. Bueno

**Affiliations:** a Millennium Institute on Immunology and Immunotherapy, Santiago, Chile; b Departamento de Genética Molecular y Microbiología, Facultad de Ciencias Biológicas, Pontificia Universidad Católica de Chile, Santiago, Chile; c Departamento de Enfermedades Infecciosas e Inmunología Pediátrica, División de Pediatría, Escuela de Medicina, Pontificia Universidad Católica de Chile, Santiago, Chile; d Centro de Investigación Clínica UC, Pontificia Universidad Católica de Chile, Santiago, Chile; e Departamento de Laboratorio Biomédico, Instituto de Salud Pública de Chile, Santiago, Chile; f Laboratorio de Virología Molecular y Celular, Programa de Virología, Instituto de Ciencias Biomédicas, Facultad de Medicina, Universidad de Chile, Santiago, Chile; g Center for Infectious Disease and Vaccine Research, La Jolla Institute for Immunology, La Jolla, California, USA; h Department of Medicine, Division of Infectious Diseases and Global Public Health, University of California, San Diego, La Jolla, California, USA; i Sinovac Biotech, Beijing, China; j Departamento de Farmacia, Facultad de Química y de Farmacia, Pontificia Universidad Católica de Chile, Santiago, Chile; k Departamento de Endocrinología, Facultad de Medicina, Escuela de Medicina, Pontificia Universidad Católica de Chile, Santiago, Chile; Albert Einstein College of Medicine

**Keywords:** CoronaVac, phase III clinical trial, SARS-CoV-2, COVID-19, booster dose

## Abstract

CoronaVac is an inactivated SARS-CoV-2 vaccine approved by the World Health Organization (WHO). Previous studies reported increased levels of neutralizing antibodies and specific T cells 2 and 4 weeks after two doses of CoronaVac; these levels were significantly reduced at 6 to 8 months after the two doses. Here, we report the effect of a booster dose of CoronaVac on the anti-SARS-CoV-2 immune response generated against the variants of concern (VOCs), Delta and Omicron, in adults participating in a phase III clinical trial in Chile. Volunteers immunized with two doses of CoronaVac in a 4-week interval received a booster dose of the same vaccine between 24 and 30 weeks after the second dose. Neutralization capacities and T cell activation against VOCs Delta and Omicron were assessed 4 weeks after the booster dose. We observed a significant increase in neutralizing antibodies 4 weeks after the booster dose. We also observed a rise in anti-SARS-CoV-2-specific CD4^+^ T cells over time, and these cells reached a peak 4 weeks after the booster dose. Furthermore, neutralizing antibodies and SARS-CoV-2-specific T cells induced by the booster showed activity against VOCs Delta and Omicron. Our results show that a booster dose of CoronaVac increases adults’ humoral and cellular anti-SARS-CoV-2 immune responses. In addition, immunity induced by a booster dose of CoronaVac is active against VOCs, suggesting adequate protection.

## INTRODUCTION

The ongoing pandemic caused by severe acute respiratory syndrome coronavirus 2 (SARS-CoV-2) has promoted the rapid development of safe, immunogenic, and effective vaccines against SARS-CoV-2 to be used by the general population, and these vaccines have successfully reduced the transmission of the disease. CoronaVac is an inactivated SARS-CoV-2 vaccine developed by Sinovac Life Sciences Co., Ltd. (Beijing, China). It is among the current vaccines approved by the World Health Organization (WHO) to combat coronavirus disease 2019 (COVID-19) and one of the most used vaccines worldwide (1, 2). Phase I and II clinical trials in China demonstrated that this vaccine induces cellular and humoral responses upon immunization (3–5). Furthermore, results from an ongoing phase III clinical trial in Chile in adults older than 18 years showed increased IgG levels with capacities and T cell-specific for SARS-CoV-2 antigens 2 and 4 weeks after the second dose of CoronaVac (6, 7). Furthermore, although the primary immunization schedule induces neutralizing antibodies in the serum of vaccinated people (8), these titers decrease in time (9) and show reduced neutralization capacities against highly transmissible variants of concern (VOCs) compared to the ancestral strain (10–13). For these reasons, booster doses were approved in August 2021 in Chile for high-risk populations and adults 20 weeks after administering the second dose. In this sense, a previous study performed in adults aged 18 to 59 years demonstrated that a booster dose of CoronaVac, applied 20 weeks after the second dose of the same vaccine, increased the levels of antibodies 3- to 5-fold compared to the levels observed 4 weeks after the second dose (14). Here, we further extend these findings by reporting the levels of neutralizing antibodies and specific T cells against SARS-CoV-2 and their activity against VOCs Delta and Omicron in adults aged ≥18 years that participated in a phase III clinical trial carried out in Chile who were vaccinated on a 28-day vaccination schedule and received a booster dose 20 weeks after the second dose.

## RESULTS

### A booster dose of CoronaVac induced a significant increase in antibody titers with neutralizing capacity in adults.

First, the safety of CoronaVac was assessed in participants previously vaccinated with two doses of the same vaccine. The first dose was inoculated between January and March 2021, the second dose was inoculated 28 ± 7 days after the first one, and the booster dose was inoculated 20 weeks ± 14 days after the second dose. In line with our previous report in the same clinical trial (5, 15), the most frequent adverse local effect was pain in the inoculation site, reported in 32.1% of the volunteers. The rest of the adverse effects were reported at a low frequency (less than 5% of the volunteers) (see Table S1 in the supplemental material). When we analyzed the data by age group, we observed that volunteers under 60 years presented more adverse effects than those older than 60 years, with more pain at the site of inoculation, induration, erythema, and swelling (see Table S1). Consistent with these findings, the first and booster doses showed that the CoronaVac vaccine has low reactogenicity and good tolerability. Most systemic adverse events were mild, as was observed with the two first doses of CoronaVac. In this sense, the adverse events more frequently reported after the booster dose were headache (19.7%), fatigue (14.2%), and myalgia (15%). Again, the volunteers under 60 years presented higher frequencies of headache and myalgias (see Table S2). These results support the notion that CoronaVac is safe and better tolerated in people over 60 years.

10.1128/mbio.01423-22.6 TABLE S1 Solicited local adverse events after inoculation in volunteers classified by arm (age group) after the booster dose. Download Table S1, DOCX file, 0.02 MB. Copyright © 2022 Schultz et al. 2022 Schultz et al. https://creativecommons.org/licenses/by/4.0/ This content is distributed under the terms of the Creative Commons Attribution 4.0 International license.

10.1128/mbio.01423-22.7 TABLE S2 Solicited systemic adverse events after inoculation in volunteers classified by arm (age group) after the booster dose. Download Table S2, DOCX file, 0.02 MB. Copyright © 2022 Schultz et al. 2022 Schultz et al. https://creativecommons.org/licenses/by/4.0/ This content is distributed under the terms of the Creative Commons Attribution 4.0 International license.

**FIG 1:**
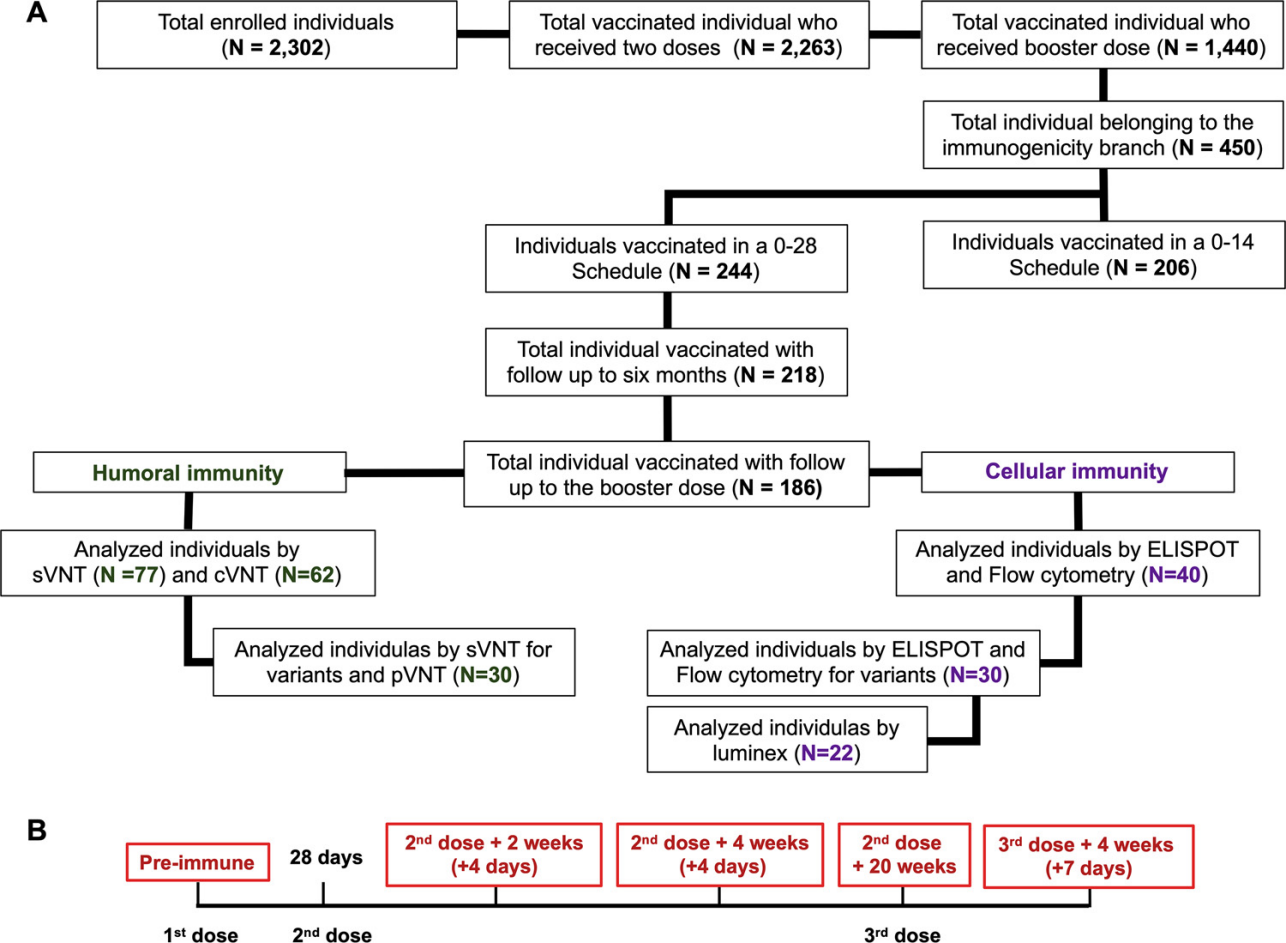
Study profile, enrolled volunteers, and cohort included in the study by 11 November 2021. (A) Of the 186 vaccinated individuals that received the booster dose, 77 that received two doses of CoronaVac in a 28-day interval (28-day schedule of vaccination) were selected from the center assigned for the immunogenicity study. Samples from the 77 volunteers were tested for neutralizing antibodies by sVNT, 62 were selected for analysis of neutralizing antibodies by cVNT, and 40 were selected for analysis of cellular immunity. Analyses for immunity against SARS-CoV-2 variants were performed on 30 volunteers for assays by use of sVNT, pVNT, and T cells. (B) Timeline of 28-day schedule of vaccination and booster dose immunization. Text in red denotes time points at which blood draws occurred and the sample collection time window.

**FIG 2:**
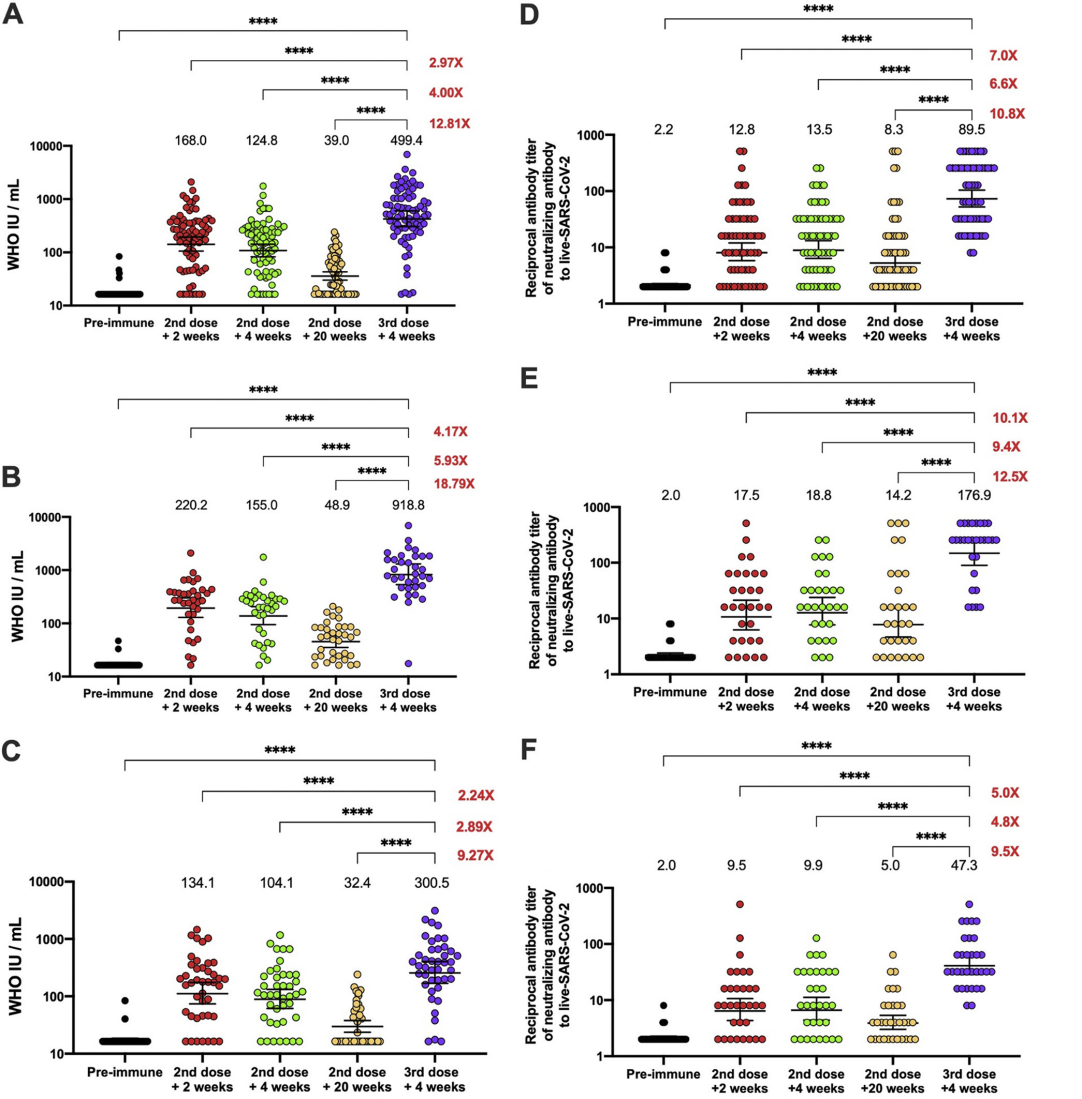
Quantification of circulating antibodies inhibiting the interaction between the S1-RBD and hACE2 and in live SARS-CoV-2 in volunteers who received the booster dose of CoronaVac. (A to C) Inhibiting antibodies were detected in the serum of volunteers immunized with CoronaVac using a surrogate viral neutralization test (sVNT), which quantified the interaction between S1-RBD and hACE2 on ELISA plates. Results were obtained from a total of 77 volunteers (A); 36 of them were adults between 18 and 59 years old (B), and 41 were ≥60 years old (C). Data is presented as WHO arbitrary units per milliliter, the numbers above each set of individual data points show the geometric mean units (GMU), the error bars indicate the 95% CI, and the number at the right represents the fold increase of the GMU 4 weeks after the third dose, compared with the respective times after administration of the second dose. (D to F) Neutralizing antibodies were detected in the serum of volunteers that received a booster dose of CoronaVac 20 weeks after the second dose, using a conventional viral neutralization test (cVNT), which quantified the reduction of cytopathic effect (CPE) in Vero E6 cells infected with SARS-CoV-2. Results were obtained from 62 volunteers (D); 30 of them were adults between 18 and 59 years old (E), and 32 of them were ≥60 years old (F). Data are expressed as the reciprocal of the highest serum dilution preventing 100% cytopathic effect, the numbers above each set of individual data points show the Geometric Mean Titer (GMT), the error bars indicate the 95% CI, and the number at the right represents the fold increase of the GMU the third dose + 4 weeks, compared with the respective times after administration of the second dose. CI were not adjusted for multiplicity and should not be used for inference. A repeated-measures one-way ANOVA assessed statistical differences to compare all times against the booster dose + 4 weeks. ****, *P* < 0.0001.

Of the 1,440 volunteers analyzed for the safety arm, 186 volunteers from the immunogenicity branch that received a booster dose of the CoronaVac were included in this study (Fig. 1A). The neutralizing capacities of serum antibodies were evaluated in 77 and 62 volunteers by surrogate virus neutralization test (sVNT) and conventional VNT (cVNT), respectively, at the five different time points indicated in Fig. 1B. As shown in Fig. 2A and D (see also Fig. S1A), the peak level of antibodies with neutralizing capacity in the total population evaluated, tested by sVNT and cVNT, was reached 2 weeks after the second dose (geometric mean units [GMU] of 168.0, 95% confidence interval [CI] of 19.5 to 34.2; geometric mean titer [GMT] of 12.8, 95% CI of 8.8 to 18.6) and 4 weeks after the second dose (GMU of 124.8, 95% CI of 96.3 to 161.7; GMT of 13.5, 95% CI of 9.6 to 19.2). However, this neutralizing capacity significantly decreased 20 weeks after the second dose (GMU of 39.0, 95% CI of 32.4 to 47.0; GMT of 8.3, 95% CI of 9.6 to 19.2), which is in line with previous reports (16, 17). Furthermore, after the booster dose, the neutralizing capacity of the antibodies increased even more than that reported 2 weeks after the second dose (GMU of 499.0, 95% CI of 370.6 to 673.0; GMT of 89.5, 95% CI of 64.0 to 125.2). Overall, we observed that 4 weeks after the booster dose, the neutralizing capacity increased more than 12-fold (sVNT) and 10-fold (cVNT) compared to the response at 20 weeks after the second dose, and it increased almost 3-fold compared to 2 weeks after the second dose (Fig. 2A and D; see also Fig. S1A).

10.1128/mbio.01423-22.2 FIG S1 Titers of antibodies inhibiting the interaction between the S1-RBD and hACE2 in volunteers that received the booster dose. Inhibiting antibodies were detected in the serum of volunteers immunized with CoronaVac by use of a surrogate viral neutralization test (sVNT), which quantified the interaction between S1-RBD and hACE2 on ELISPOT plates. Results were obtained from a total of 77 volunteers (A); 36 of them were adults between 18 and 59 years (B) and 41 of them were adults ≥60 years old (C). Data are presented as the logarithm of the reciprocal antibody titer regarding the time after the first dose. The numbers above the bars show the geometric mean titer (GMT), and the error bars indicate the 95% CI. A repeated-measures one-way ANOVA assessed statistical differences to compare all times against the booster dose + 4 weeks. **, *P* < 0.005; ****, *P* < 0.0001. Download FIG S1, TIF file, 0.9 MB. Copyright © 2022 Schultz et al. 2022 Schultz et al. https://creativecommons.org/licenses/by/4.0/ This content is distributed under the terms of the Creative Commons Attribution 4.0 International license.

In adults 18 to 59 years old, the neutralizing capacity of circulating antibodies tested by sVNT and cVNT (Fig. 2B and E; see also Fig. S1B) reached high titers 4 weeks after the booster dose (GMU of 918.8, 95% CI of 623.4 to 1,354; GMT of 176.9, 95% CI of 111.7 to 280.1), increasing more than 18- and 12-fold compared to 20 weeks after the second dose (GMU of 48.9, 95% CI of 37.6 to 63.5; GMT of 14.2, 95% CI of 7.1 to 28.4) and more than 4-fold compared to 2 weeks after the second dose (GMU of 220.2, 95% CI of 150.7 to 321.7; GMT of 17.5, 95% CI of 9.8 to 31.3) (Fig. 2B and E; see also Fig. S1B). The seropositivity rate in this group reached 100% 4 weeks after the booster dose (Table 1). On the other hand, 53.2% of the total volunteers were adults ≥60 years old. In this group, the same tendency was observed, as seen in Fig. 2C and F and Fig. S1C, with an increase in the level of neutralizing antibodies evaluated by both techniques of more than 9-fold at 4 weeks after the booster dose (GMU of 300.5, 95% CI of 203.5 to 443.6; GMT of 47.3, 95% CI of 32.1 to 69.5) compared to the response observed 20 weeks after the second dose (GMU of 32.4, 95% CI of 25.1 to 41.8; GMT of 5.0, 95% CI of 3.5 to 7.0).

**TABLE 1:** Seropositivity rates, GMT, and GMU of circulating neutralizing antibodies against SARS-CoV-2 RBD^*a*^

Testing method	Age group (yrs)	Indicator	2nd dose + 2 wks	2nd dose + 4 wks	2nd dose + 20 wks	3rd dose + 4 wks
sVNT	All	Seropositivity [no. positive/total no. tested (%)]	72/77 (93.5)	73/77 (94.8)	38/77 (49.4)	75/77 (97.4)
		GMU (95% CI)	168.0 (126.8–222.5)	124.8 (96.3–161.7)	39.0 (32.4–47.0)	499.4 (370.6–673.0)
		GMT (95% CI)	25.8 (19.5–34.2)	16.6 (13.1–21.0)	3.5 (3.0–4.1)	53.0 (40.8–68.8)
	18–59	Seropositivity [no. positive/total no. tested (%)]	35/36 (97.2)	36/36 (97.2)	24/36 (66.7)	36/36 (100)
		GMU (95% CI)	220.2 (150.7–321.7)	155.0 (108.0–222.6)	48.9 (37.6–63.5)	918.8 (623.4–1,354)
		GMT (95% CI)	33.3 (23.4–47.3)	19.1 (14.0–26.1)	4.3 (3.4–5.4)	82.8 (59.7–114.8)
	≥60	Seropositivity [no. positive/total no. tested (%)]	38/41 (90.5)	39/42 (92.9)	15/42 (35.7)	40/42 (95.2)
		GMU (95% CI)	134.1 (89.2–201.6)	104.1 (71.8–151.0)	32.4 (25.1–41.8)	300.5 (203.5–443.6)
		GMT (95% CI)	20.8 (13.6–31.9)	14.7 (10.3–21.0)	2.4 (2.4–3.5)	36.5 (25.3–52.7)
cVNT	All	Seropositivity [no. positive/total no. tested (%)]	49/62 (79.0)	51/62 (82.3)	44/62 (71.0)	62/62 (100)
		GMT (95% CI)	12.8 (8.8–18.5)	13.5 (9.6–19.2)	8.3 (5.6–12.2)	89.5 (64.0–125.2)
	18–59	Seropositivity [no. positive/total no. tested (%)]	25/30 (83.3)	27/30 (90.0)	23/30 (76.7)	30/30 (100)
		GMT (95% CI)	17.5 (9.8–31.3)	18.8 (11.2–31.7)	14.2 (7.1–28.4)	176.9 (111.7–280.1)
	≥60	Seropositivity [no. positive/total no. tested (%)]	24/32 (75.0)	24/32 (75.0)	21/32 (65.6)	32/32 (100)

		GMT (95% CI)	9.5 (5.8–15.4)	9.9 (6.2–15.8)	5.0 (3.5–7.0)	47.3 (32.1–69.5)

a Samples from the 77 volunteers were used to evaluate the antibodies with neutralizing capacity at the different visits by use of either sVNT or cVNT.

The seropositivity rate in this age group reached 100% 4 weeks after the booster dose (Table 1). In addition, the seropositivity rate achieved at 4 weeks after the booster dose was the highest compared with the other visits in this study in the total vaccinated group and both groups analyzed.

### A booster dose of CoronaVac induced a robust cellular immune response in adults.

**FIG 3:**
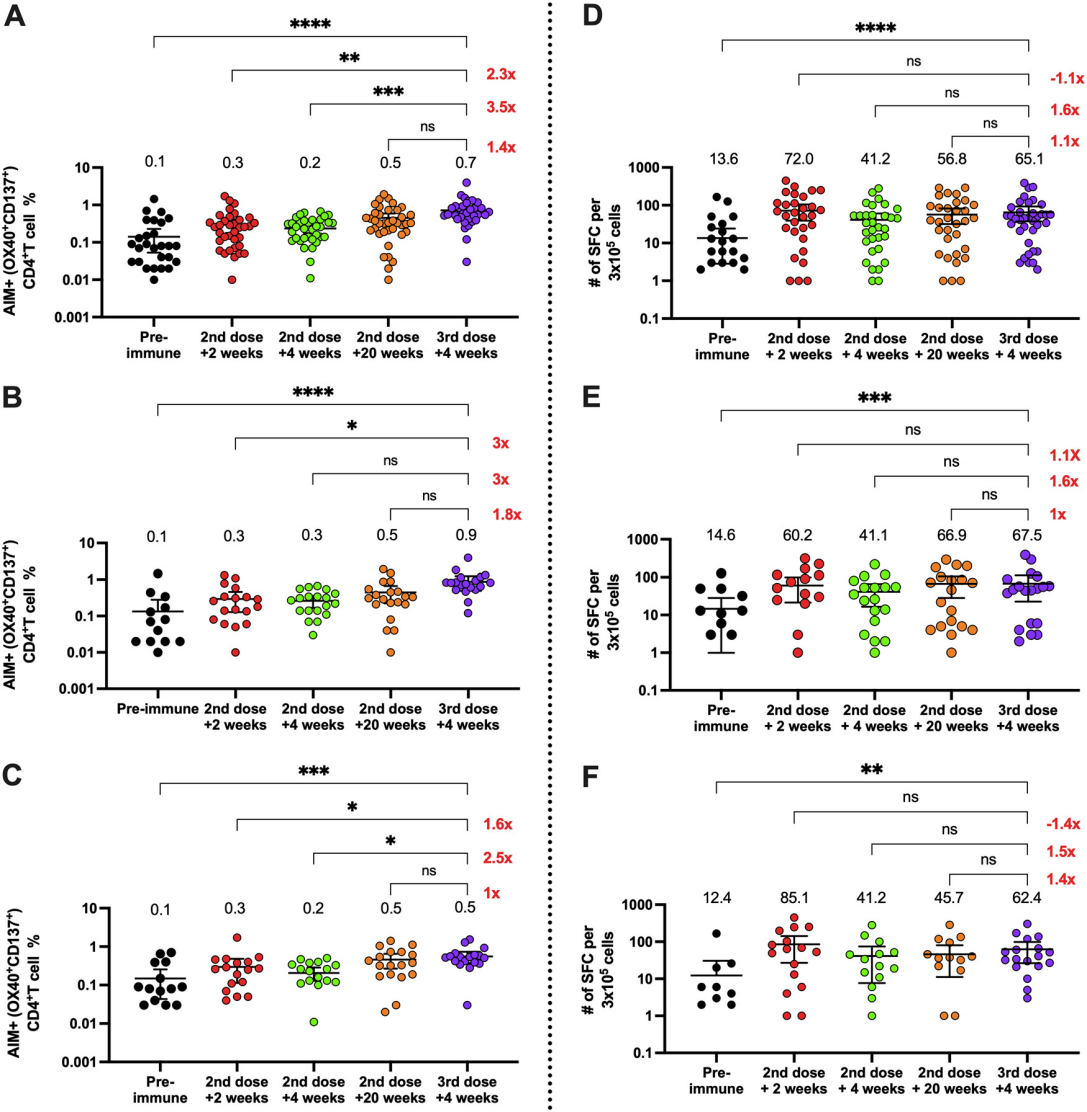
Changes in activation-induced marker (AIM) expression in CD4^+^ T cells and in the number of IFN-γ-secreting cells specific for SARS-CoV-2 after a booster dose of CoronaVac. (A to C) AIM^+^ CD4^+^ T cells were quantified in peripheral blood mononuclear cells of volunteers that received a booster dose of CoronaVac 20 weeks after the second dose by flow cytometry upon stimulation with megapools of peptides derived from SARS-CoV-2 proteins. The percentages of activated AIM^+^ CD4^+^ T cells (OX40^+^ CD137^+^) were determined upon stimulation for 24 h with MP-S + R in samples obtained at preimmune, 2 weeks after the second dose, 4 weeks after the second dose, 20 weeks after the second dose, and 4 weeks after the booster dose. Data from flow cytometry were normalized against DMSO and analyzed separately by a Friedman test against the booster dose. Results were obtained from a total of 40 volunteers (A); 21 of them were adults between 18 and 59 years old (B), and 19 of them were ≥60 years old (C). Changes in the secretion of IFN-γ were quantified as the number of spot-forming cells (SFCs) in peripheral blood mononuclear cells of volunteers that received a booster dose of CoronaVac 20 weeks after the second dose. (D to F) Data were obtained upon stimulation with MP-S + R for 48 h in samples obtained at preimmune, 2 weeks after the second dose, 4 weeks after the second dose, 20 weeks after the second dose, and 4 weeks after the booster dose. Results were obtained from a total of 40 volunteers (D); 21 of them were adults between 18 and 59 years old (E), and 19 of them were ≥60 years old (F). The number at the right represents the fold increase of the GMU 4 weeks after the booster dose compared with the respective times after administering the second dose. Data from ELISPOT were analyzed separately by Friedman test against the booster dose. *, *P* < 0.05; **, *P* < 0.005; ***, *P* < 0.001; ****, *P* < 0.0001.

The cellular responses following a booster dose of CoronaVac were evaluated in 40 volunteers. We observed that CD4^+^ T cell activation was increased 20 weeks after the second dose compared to the other time points in both age groups, suggesting that CoronaVac can stimulate CD4^+^ T cell responses sustained over time (Fig. 3A to C). Importantly, we observed a significant increase in CD4^+^ T cell activation in both groups following the booster dose compared to the preimmune sample and samples obtained 2 and 4 weeks after the second dose (Fig. 3A to C). However, only the participants older than 60 years exhibited a significant increase compared to the time point at 4 weeks after the second dose. Participants aged 18 to 59 years also exhibited an increase in anti-SARS-CoV-2 activation-induced marker-positive (AIM^+^) CD4^+^ T cells compared to this time point, but the increase was not significant, which may have been due to the data dispersion. Indeed, the mean number of AIM^+^ CD4^+^ T cells was higher in the age group 18 to 59 years, and so it may be misleading to suggest that only participants older than 60 years were more responsive to the booster dose.

Moreover, we did not observe a significant increase in the expression of AIM by CD8^+^ T cells following the booster, suggesting that specific CD8^+^ T cell responses induced by CoronaVac are not detected with the current methodologies, even after a third dose (see Fig. S2A and C). Accordingly, we observed an increase in gamma interferon (IFN-γ) production upon stimulation with megapools of peptides (MPs) S and R by enzyme-linked immunosorbent spot assay (ELISPOT) 4 weeks after the booster dose for both groups, compared to the preimmune sample (Fig. 3D to F). As with the flow cytometry results, we did not observe a significant increase of IFN-γ spot-forming cells (SFCs) upon stimulation with CD8 MPs at any time point (see Fig. S2). These results suggest that although humoral responses decrease over time following vaccination with CoronaVac, anti-SARS-CoV-2 CD4^+^ T cell responses remain significantly increased compared to preimmune samples, and the booster dose promotes slight increases in both IFN-γ production and CD4^+^ T cell activation that are not significantly different from the levels observed 20 weeks after the second dose.

10.1128/mbio.01423-22.3 FIG S2 Changes in activation-induced marker (AIM) expression in CD8^+^ T cells through flow cytometry and the number of IFN-γ-secreting cells upon stimulation with megapools of peptides derived from SARS-CoV-2, measured after the booster dose of CoronaVac. AIM^+^ CD8^+^ T cells were quantified in peripheral blood mononuclear cells of volunteers that received a booster dose of CoronaVac 20 weeks after the second dose by flow cytometry upon stimulation with megapools of peptides derived from SARS-CoV-2 proteins. The percentages of activated AIM^+^ CD8^+^ T cells (CD69^+^ CD137^+^) were determined upon stimulation for 24 h with MP-S + R in samples obtained at preimmune, 2 weeks after the second dose, 4 weeks after the second dose, 20 weeks after the second dose, and 4 weeks after the booster dose. Data from flow cytometry were normalized against DMSO and analyzed separately by a Friedman test against the booster dose. Results were obtained from a total of 40 volunteers (A); 21 of them were adults between 18 and 59 years (B), and 19 of them were ≥60 years old (C). Changes in the secretion of IFN-γ were quantified as the number of spot-forming cells (SFCs) in peripheral blood mononuclear cells of volunteers that received a booster dose of CoronaVac 20 weeks after the second dose. (D to F) Data obtained upon stimulation with MP-S + R for 48 h in samples obtained at preimmune, 2 weeks after the second dose, 4 weeks after the second dose, 20 weeks after the second dose, and 4 weeks after the booster dose. Results were obtained from a total of 40 volunteers (D); 21 of them were adults between 18 and 59 years (E), and 19 of them were ≥60 years old (F). Data from ELISPOT were analyzed separately by Friedman test against the booster dose, with no statistical differences. Download FIG S2, TIF file, 1.5 MB. Copyright © 2022 Schultz et al. 2022 Schultz et al. https://creativecommons.org/licenses/by/4.0/ This content is distributed under the terms of the Creative Commons Attribution 4.0 International license.

### Neutralizing antibodies and specific T cells induced by a booster dose of CoronaVac recognize Delta and Omicron variants of SARS-CoV-2.

**FIG 4:**
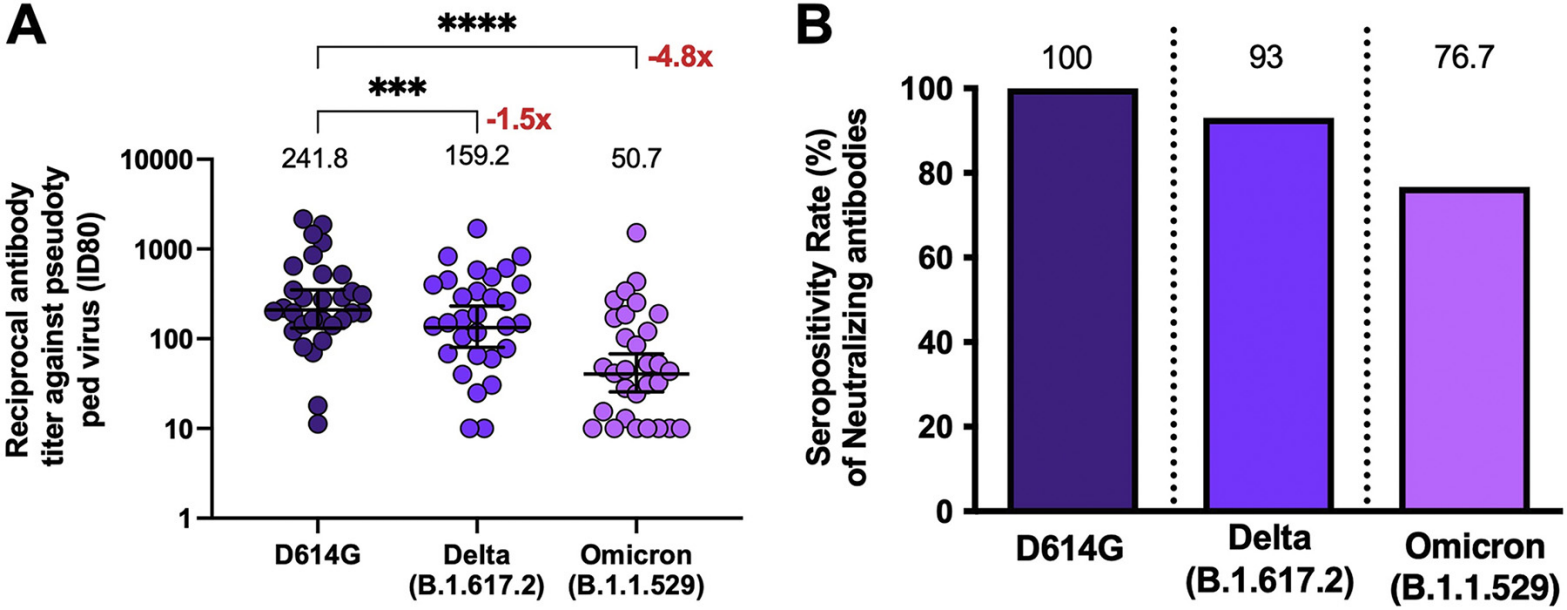
Quantification of circulating neutralizing antibodies against SARS-CoV-2 variants in volunteers that received the booster dose of CoronaVac. (A) Neutralizing antibodies were detected in the serum of 30 volunteers at 4 weeks after the booster dose of CoronaVac, by use of a pseudotyped virus neutralization test (pVNT). Data are expressed as the reciprocal of the highest dilution preventing 80% of the infection (ID_80_). The numbers above the bars show the means, and the error bars indicate the 95% CI. The number at the right represents the fold decrease of the GMT 4 weeks after the booster dose, compared with the response of D614G. (B) Seropositivity rate of neutralizing antibodies for each time point analyzed. The numbers above the bars show the percent seropositivity rates in the respective graphs. The number at the right represents the fold increase of the GMU 4 weeks after the third dose, compared with the respective times after administering the second dose. A repeated measures one-way ANOVA assessed statistical differences of the GMT to compare each variant against D614G. *, *P* < 0.05; ***, *P* < 0.001; ****, *P* < 0.0001.

As we observed that the neutralization capacity increased significantly with the booster dose and, knowing that vaccinated volunteers exhibit decreased neutralization against VOC (10), we proceeded to evaluate the neutralizing abilities in 30 booster-vaccinated individuals by using a pseudotyped virus neutralization test (pVNT) assay against two VOCs of SARS-CoV-2 and comparing these results with the level obtained for the SARS-CoV-2 mutant with a D-to-G mutation at position 614 (D614G) (Fig. 4A and B). We observed that the titers of antibodies with neutralizing capacities against the Delta and Omicron variants showed a reduction compared to the levels achieved for the D614G variant (D614G: GMT of 241.8, CI of 155.7 to 375.6; Delta: GMT of 159.2, CI of 99.1 to 256.0; Omicron: GMT of 50.7, CI of 30.4 to 84.8), with a reduction of 1.5 for Delta and 4.8 for Omicron, which was statistically significant for the latter (Fig. 4A). However, when we compared the changes in seropositivity for Delta and Omicron (Fig. 4B), we observed rates of 93% and 76.7%, respectively, following the booster dose (Table 2). Furthermore, neutralization assays against the Delta variant with a cVNT in a different group of 19 volunteers also showed that antibodies induced 4 weeks after the booster dose had a reduced capacity to neutralize this VOC (see Fig. S3A). However, the seropositivity rate observed was 84% (see Fig. S3B).

**TABLE 2:** Seropositivity rates and GMT of circulating neutralizing antibodies against SARS-CoV-2 RBD of D614G and variants of concern (Delta and Omicron)^*a*^

Variant	D614G	Delta (B.1.617.2)	Omicron (B.1.1.529)
Indicators	3rd dose + 4 wks	3rd dose + 4 wks	3rd dose + 4 wks
Seropositivity [no. positive/total no. tested (%)]	30/30 (100)	28/30 (93.3)	23/30 (76.6)
GMT (95% CI)	241.8 (155.7–375.6)	159.2 (99.1–256.0)	50.7 (30.4–84.8)

a Samples from 30 volunteers were evaluated for antibodies with neutralizing capacity against D614G and VOCs by pVNT.

10.1128/mbio.01423-22.4 FIG S3 Quantification of circulating neutralizing antibodies against live SARS-CoV-2 variants in volunteers that received the booster dose of CoronaVac. (A) Neutralizing antibodies were detected in the serum of 19 volunteers that received a booster dose of CoronaVac 20 weeks after the second dose, using a conventional Viral Neutralization Test (cVNT). Data are expressed as the reciprocal of the highest serum dilution preventing 100% cytopathic effect (CPE) in Vero E6 cells infected with SARS-CoV-2 variants D614G and Delta (Chilean isolates). The numbers above each set of individual data points show the geometric mean titer (GMT), and the error bars indicate the 95% CI. (B) Seropositivity rate of neutralizing antibodies is shown for each group analyzed. The numbers above the bars show the percentage of seropositivity rate in the respective graphs. A Wilcoxon *t* test was performed to assess statistical differences of the GMT to compare between groups. *, *P* < 0.05; ***, *P* < 0.001. Download FIG S3, TIF file, 0.9 MB. Copyright © 2022 Schultz et al. 2022 Schultz et al. https://creativecommons.org/licenses/by/4.0/ This content is distributed under the terms of the Creative Commons Attribution 4.0 International license.

**FIG 5:**
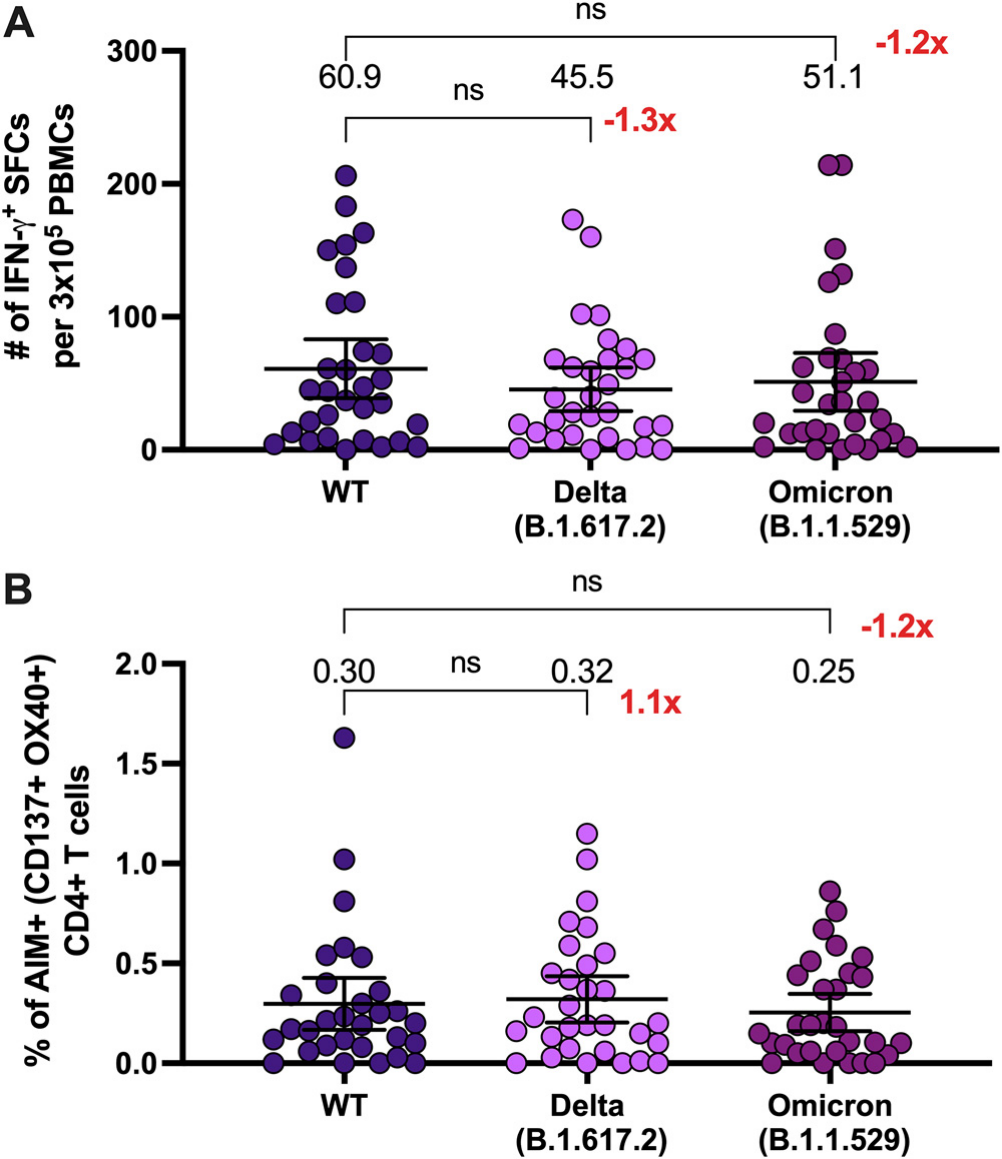
A booster dose of CoronaVac induces changes in the number of IFN-γ-secreting cells and in activation-induced marker (AIM) expression in CD4^+^ T cells specific for the Spike protein of SARS-CoV-2 variants. (A) Changes in the secretion of IFN-γ were determined as the number of spot-forming cells (SFCs). Data were obtained upon stimulation of PBMC with MP-S of variants of concern of SARS-CoV-2 for 48 h in samples obtained 4 weeks after the booster dose. Data shown represent means + 95% CI. Data from 30 volunteers were analyzed 4 weeks after the booster dose to compare the MP-S of the variants of concern. Data from ELISPOT were analyzed separately by Friedman test against the WT MP-S. No significant differences were obtained. (B) AIM^+^ CD4^+^ T cells were quantified in peripheral blood mononuclear cells of 30 volunteers 4 weeks after they received a booster dose of CoronaVac by use of flow cytometry, upon stimulation with megapools of peptides derived from proteins of variants of concern of SARS-CoV-2. The percentage of activated AIM^+^ CD4^+^ T cells (OX40^+^ CD137^+^) was determined after stimulation for 24 h with MP-S + R in samples obtained 4 weeks after the booster dose. The number at the right represents the fold increase of the GMU 4 weeks after the third dose, compared with the respective times after administering the second dose. Data shown represent means + 95% CI. Data from flow cytometry were normalized against DMSO. No significant differences were obtained between WT and the variant MP stimulation.

The cellular responses to VOCs following a booster dose of CoronaVac were also evaluated in 30 volunteers using MPs of peptides derived from the Spike protein of Delta and Omicron variants. We observed equivalent numbers of IFN-γ-secreting T cells after 4 weeks of the booster dose upon stimulation with MP-S of SARS-CoV-2 wild type (WT), Delta, or Omicron variant (Fig. 5A), with no significant differences between the responses against the MP-S of the variants compared to the MP-S of the WT strain. AIM^+^ CD4^+^ T cells were also analyzed in these samples, and no differences were observed (Fig. 5B). We also quantified the production of different cytokines in the supernatants of peripheral blood mononuclear cells (PBMCs) stimulated with the MP-S of WT, Delta, and Omicron variants, and we observed that at 4 weeks after the booster dose, the stimulated cells secreted equivalent levels of interleukin 2 (IL-2) and IFN-γ (see Fig. S4). These results suggest that although the humoral response, measured as neutralization capacities and seroconversion against these VOCs, is lower than the humoral response against the D614G strain, the cellular responses against SARS-CoV-2 VOCs are equivalent to the responses elicited by the ancestral strain in volunteers vaccinated with a booster dose.

10.1128/mbio.01423-22.5 FIG S4 IL-2 and IFN-γ secretion are induced in PBMC stimulated with megapools (MP) of variants of SARS-CoV-2 4 weeks after the booster dose with CoronaVac. Luminex was used to determine changes in cytokine secretion. Data were obtained upon stimulation of PBMCs with MP against protein S of WT, Delta, and Omicron variant of SARS-CoV-2 for 20 h in samples obtained 4 weeks after the booster dose. Results were obtained from a total of 20 volunteers for IL-2 secretion (A) and IFN-γ (B). Data represent means + 95% CI. A repeated-measures one-way ANOVA assessed statistical differences of the GMT to compare each variant against D614G. Download FIG S4, TIF file, 0.9 MB. Copyright © 2022 Schultz et al. 2022 Schultz et al. https://creativecommons.org/licenses/by/4.0/ This content is distributed under the terms of the Creative Commons Attribution 4.0 International license.

## DISCUSSION

In this study, we evaluated the humoral and cellular immune responses generated 4 weeks after the application of a booster dose of inactivated CoronaVac vaccine in a cohort of volunteers enrolled in a phase III clinical trial held in Chile. The data reported here showed that, although there was an adequate humoral response after two doses of CoronaVac, with 65.9% effectiveness in preventing COVID-19 (7), both the sVNT and cVNT assays showed a decrease in the GMT of neutralizing capacities of circulating antibodies against SARS-CoV-2 20 weeks after the second dose (Fig. 2). Due to this decrease in neutralizing capacities, a booster dose of CoronaVac was evaluated in a clinical study in China, which showed promising results in enhanced humoral immune responses (14, 18). Data reported here show that after the booster dose, the neutralizing titers and seroconversion rates increased in the whole group, to a higher extent than 2 weeks after the second dose, where the peak in neutralization was previously observed, which is in line with the observations of Clemens et al. (11). Also, we observed a steady activation of the CD4^+^ T cells and secretion of IFN-γ in response to SARS-CoV-2 peptide MPs at the time points evaluated (Fig. 3).

Since the neutralizing antibody titers correlated with protection against SARS-CoV-2 infection (8), these results likely imply a better outcome and protection against COVID-19, as reported in previous studies performed in Israel, which showed a decrease in the transmission and the disease severity of this virus 12 or more days after booster inoculation (19). In Chile, the effectiveness and prevention of hospitalization increased when assessed 14 days after the booster dose of CoronaVac (20). Another study, performed with a booster dose of CoronaVac, showed that an additional dose resulted in good neutralization capacity against parental SARS-CoV-2 and the Delta variant 4 weeks after the booster dose, generating a long-lasting humoral response that was due to an enhancement of the memory immune response generated by B cells (18).

Adults ≥60 years old produced lower levels of antibodies with neutralizing capacities than the whole group during this study (Fig. 2C and F), which was also described previously (5). In this sense, our results are equivalent to those described in phases I and II of the clinical trial performed with CoronaVac in China, showing that the neutralizing antibody titers in this group decreased 5 months after the second dose and that a booster dose was required 6 to 8 months after the first vaccination to rapidly increase and maintain the neutralizing antibody titers (21).

In terms of the T cell response (Fig. 3), other studies have shown that Pfizer BNT162b2 and mRNA-1273 induce durable anti-SARS-CoV-2 CD4^+^ T cell activation and cytokine production up to 6 months following vaccination. However, it remains to be elucidated whether the expression of AIM by CD4^+^ T cells and cytokine production increase after a booster dose with these vaccines (22, 23). Here, we observed that the activation of CD4^+^ T cells and IFN-γ production stayed increased up to 20 weeks after the second dose, and that after the booster dose, both parameters increased in the age group 18 to 59 years old and were maintained at the levels observed 20 weeks after the second dose in adults ≥60 years old. In contrast to BNT162b2 and mRNA-1273 vaccines, CoronaVac delivers the Spike protein upon immunization and other viral antigens, which may explain why vaccinated individuals still display AIM^+^ CD4^+^ T cells 5 months after the second dose, regardless of a third dose. In addition, our data indicate that volunteers vaccinated with CoronaVac also exhibit anti-SARS-CoV-2 CD4^+^ T cell responses against other proteins from the virus different from the Spike protein, and this may confer an advantage compared to other vaccine platforms that only target anti-Spike immune responses. Further assays are required to evaluate immune responses against other SARS-CoV-2 proteins, such as M, N, and E proteins, which are included in the inactivated viral particle contained in the CoronoVac vaccine.

Although we did not detect a significant increase in AIM^+^ CD8^+^ T cells or IFN-γ production upon stimulation with CD8 MPs, other studies have reported increased anti-SARS-CoV-2-CD8^+^ T cell responses in volunteers vaccinated with CoronaVac, which could have been due to stimulation with different peptides or proteins and the evaluation of other parameters, such as granzyme production (24, 25). Indeed, we observed high levels of IFN-γ production and a high frequency of anti-SARS-CoV-2 AIM^+^ CD8^+^ T cells in the preimmune samples of all participants, suggesting some nonspecific responses in our assays. Therefore, we cannot rule out that the participants vaccinated with a booster dose may exhibit enhanced anti-SARS-CoV-2-CD8^+^ T cell responses, but additional assays may be required to support this hypothesis. On the other hand, heterologous vaccination combining adenovirus-vectored and mRNA vaccines may enhance Th1 CD4^+^ and CD8^+^ T cell responses against SARS-CoV-2 (26). However, it remains to be elucidated whether a heterologous vaccination with two doses of CoronaVac and a booster with another vaccine may generate higher and more durable T cell responses than homologous vaccination.

When the neutralization capacity analyzed using pVNT of the VOCs Delta and Omicron was evaluated 4 weeks after the booster dose, we observed differences in the neutralization capacity compared to that for D614G, which does not exhibit mutations in the receptor binding domain (RBD) of the S1 protein (Fig. 4 and Table 2). We previously reported that CoronaVac could induce neutralization against the Delta variant 4 weeks after the second dose, although to a lesser extent than the the response to the WT strain (10). Although we did not observe similar levels of enhanced neutralization against the Delta variant after the booster dose based on pVNT (Fig. 4A), the seropositivity against the Delta variant was almost 100% (Fig. 4B and Table S4), which is in line with findings of previous studies (18, 27). Here, we also showed that a booster dose induced neutralization against the Omicron variant, which has rapidly spread worldwide and is the predominant circulating variant to date (28). The high number of mutations described for the RBD of this variant has been associated with increased evasion of neutralizing responses in either unvaccinated or vaccinated subjects (28). Although the neutralization observed in subjects vaccinated with a booster dose of CoronaVac was significantly lower than that observed for the D614G variant, we observed a seropositivity of 76.7% following the booster dose, suggesting some degree of protection in most of the vaccinees. In this sense, it has been reported that a heterologous vaccination schedule may induce a higher neutralization ability and a better neutralization against variants of concern such as Delta (29) and Omicron (12). Similarly, a comparison between heterologous and homologous booster schedules after vaccination with CoronaVac showed an increase in neutralization against the VOCs Delta and Omicron (11). There are discrepancies between the results in neutralization titers, which can be attributed to the neutralization assays performed and/or the study population; however, important booster responses were observed in these studies, and seropositivity reached after the booster dose of CoronaVac against VOCs were also similar (12). Of note, we could not perform cVNT to assess neutralization against the Omicron variant, and further assays need to be performed to address this limitation.

10.1128/mbio.01423-22.9 TABLE S4 Seropositivity rates and GMT of circulating neutralizing antibodies against SARS-CoV-2 RBD of D614G and the Delta variant. Download Table S4, DOCX file, 0.02 MB. Copyright © 2022 Schultz et al. 2022 Schultz et al. https://creativecommons.org/licenses/by/4.0/ This content is distributed under the terms of the Creative Commons Attribution 4.0 International license.

In the case of the cellular response, we characterized CD4^+^ T cell responses following a booster dose of CoronaVac against the Omicron variant of SARS-CoV-2. Previous studies using the same MP from VOCs evaluated here have shown that CD4^+^ T cells respond to VOCs to a similar extent as with the ancestral strain in individuals vaccinated with CoronaVac (10, 30) and mRNA vaccines, which has been explained by the high conservation of T cell epitopes. In this sense, the booster dose of CoronaVac induces the expression of CD4^+^ T cell activation markers and secretion of IFN-γ and IL-2 against the VOCs Delta and Omicron, comparable to the response generated against the WT strain (Fig. 5). In line with this, a recent study showed that T cell responses against the ancestral strain are cross-reactive against the Omicron variant in convalescent individuals and volunteers vaccinated with Pfizer BNT162b2 (31), supporting the idea that the induction of T cell responses against the ancestral strain may be protective against the Omicron variant. Indeed, studies in subjects vaccinated with Pfizer BNT162b2, Moderna mRNA-1273, Ad26.COV2.S, or NVX-CoV2373 also have shown lower levels of neutralizing responses and memory B cells against Omicron compared to the ancestral strain, even after a booster dose, but comparable T cell responses were reported against Omicron compared to the ancestral strain (32, 33). Similarly, although memory B cell responses against Omicron wane over time in subjects vaccinated with two doses of mRNA vaccines, T cell responses against VOCs, including Omicron, are maintained up to 6 months postvaccination (33). These findings suggest that T cell responses against Omicron may even compensate for the lower levels of neutralizing antibodies in vaccinated subjects, which may also be the case for participants vaccinated with CoronaVac.

Our report shows that the booster dose of CoronaVac in a 28-day schedule induces antibodies with neutralizing capacities that are higher than the levels observed at 2 and 4 weeks after the second dose, generating an increased humoral response even in adults ≥60 years old. Additionally, our results suggest that the third dose of CoronaVac supports CD4^+^ T cell activation, which may confer either protection or enhanced immune responses against the virus and prevent severe disease following exposure to SARS-CoV-2. Notably, the humoral and cellular immune responses promoted by a booster dose of CoronaVac show activity against Delta and Omicron variants and probably result in better effectiveness of this vaccine during the predominance of these VOCs.

Finally, among the advantages of this inactivated vaccine are the easy preparation method, transport, and storage, especially in countries with low incomes. Also, as we mentioned here, the vaccine does not induce unexpected side effects, with only mild local and systemic adverse effects. Our data indicate that the inactivated vaccine CoronaVac induces both humoral and cellular immune responses against SARS-CoV-2. Considering that the vaccine includes peptides from all the different antigens reported for SARS-CoV-2, it is important to study the immune response generated against viral proteins other than the Spike protein. This type of vaccine may confer an advantage compared to other vaccine platforms that only target anti-Spike immune responses.

### Strengths.

This work further characterizes immune responses induced by two doses of CoronaVac separated by 4 weeks and a booster dose 5 months after the second dose in healthy adults from the Chilean population. We evaluated four different time points following vaccination (2 weeks after the second dose, 4 weeks after the second dose, 5 months after the second dose, and 4 weeks after the booster dose), thus providing a complete picture of the durability of immune responses elicited by CoronaVac. The vaccination with CoronaVac in a 28-day schedule is safe and well tolerable and does not present important secondary effects, as the local and systemic adverse events are mild. Also, we report increased antibodies with neutralizing capacities following the booster dose compared to the levels observed 5 months after the second dose, which were evaluated using a surrogate neutralization assay and expressed in WHO arbitrary units, allowing their comparison to other SARS-CoV-2 vaccines. We confirmed this increased neutralization by using a conventional neutralization assay. Remarkably, the booster dose also enhanced CD4^+^ T cell responses upon stimulation with megapools of peptides from the proteome of SARS-CoV-2, increasing IFN-γ secretion and the expression of activation-induced markers. In addition, we found that the booster dose induced reduced neutralization against the Delta and Omicron variants compared to that against the D614G mutant, as measured in a pseudovirus neutralization assay. In contrast, IFN-γ secretion and T cell activation against these variants of concern were similar in comparison with the WT strain. Therefore, a third dose of CoronaVac in a homologous vaccination schedule improves its immunogenicity in healthy volunteers. It is important that the immune response generated by an inactivated vaccine, performed with the whole virus, can induce an immune response (humoral or cellular) to viral proteins different from the Spike proteins, and this may confer an advantage compared to other vaccine platforms that only target anti-Spike immune responses.

### Limitations.

This study has several limitations, such as the reduced sample size for the assays and the absence of data for neutralization against the Omicron variant obtained with a conventional viral neutralization test. The assessment of total antibody response against Spike proteins and other SARS-CoV-2 proteins would also add additional information about the humoral immune response against SARS-CoV-2 after the booster dose. Due to the limit of quantification of the technique, samples with an undetermined concentration at the lowest dilution tested (1:4) were assigned the lower limit of quantification (16.4 IU). Also, it is necessary to analyze more time after the booster dose, as the response 4 weeks after the booster dose is short for evaluation if there is a maintained or decreased immune response measured as neutralizing antibody capacities or in the cellular response as we observed previously 6 months after the first dose, which was the reason to use this booster dose. Further studies are necessary in order to evaluate if for this homologous schedule another booster dose is necessary.

## MATERIALS AND METHODS

### Volunteers and sample collection.

Blood samples were obtained from volunteers recruited in the clinical trial CoronaVac03CL (clinicaltrials.gov NCT04651790) carried out in Chile starting in November 2020. The Institutional Scientific Ethical Committee of Health Sciences reviewed and approved the study protocol at the Pontificia Universidad Católica de Chile (number 200708006). Trial execution was approved by the Chilean Public Health Institute (24204/20) and was conducted according to the current Tripartite Guidelines for Good Clinical Practices, the Declaration of Helsinki (34), and local regulations. A complete list of inclusion and exclusion criteria has been reported (5). The frequency of local and systemic adverse effects (AEs) occurring 7 days after the booster dose by age group (ages 18 to 59 and ≥60 years) was evaluated in 1,440 volunteers that received the booster dose (see Tables S1 and S2 in the supplemental material). These data are in accordance with results previously reported (5, 15). Of these 1,440 volunteers, on 11 November 2021 186 volunteers from the immunogenicity branch were analyzed. The antibody- and cell-mediated immune responses were evaluated in volunteers who had completed their previous visits in one of the study centers (Fig. 1A). Demographic and clinical data from these volunteers are presented in Table 3. The selection of these volunteers was not biased due to their immune responses before the booster, and samples were evaluated by arrival order. Blood samples were obtained from all the volunteers before administration of the first dose (preimmune), 2 weeks after the second dose (+4 days), 4 weeks after the second dose (+4 days), 20 weeks (or 5 months) after the second dose (or booster dose), and 4 weeks (+7 days) after the booster dose (Fig. 1B). The breakthrough cases (19 cases after received the booster dose) were followed as part of the phase III clinical trial. However, they are not included in this study (6).

**TABLE 3:** Demographic and comorbidity data for the 77 volunteers

Sex and age group	Total no. (%)^*a*^	No. (% of all volunteers) with comorbidity^*b*^						
		AHT	AR	MD	Obesity	Insulin resistance	COPD	HT
Female								
All	41 (53.2)	11 (14.3)	8 (10.4)	1 (1.3)	6 (7.8)	6 (7.8)	3 (3.9)	7 (9.1)
18–59 yrs	18 (23.4)	3 (3.9)	6 (7.8)	0	2 (2.6)	3 (3.9)	0	2 (2.6)
≥60 yrs	23 (30.0)	8 (10.4)	2 (2.6)	1 (1.3)	4 (5.2)	3 (3.9)	3 (3.9)	5 (6.5)
Male								
All	36 (46.8)	11 (14.3)	8 (10.4)	3 (3.9)	11 (14.3)	1 (1.3)	0	1 (1.3)
18–59 yrs	17 (22.0)	4 (5.2)	4 (5.2)	2 (2.6)	4 (5.2)	0	0	0
≥60 yrs	19 (24.6)	7 (9.1)	4 (5.2)	1 (1.3)	7 (9.1)	1 (1.3)	0	1 (1.3)

a Percentages are per all 77 volunteers with the characteristic.

b AHT, arterial hypertension; COPD, chronic obstructive pulmonary disease; MD, mellitus diabetes; HT, hypothyroidism; AR, allergic rhinitis.

### Procedures.

The presence of antibodies against RBD with neutralizing capacities was measured in sera from volunteers that had completed all their study visits, including 1 month after the booster dose of CoronaVac. The neutralizing capacities of circulating antibodies were evaluated by a surrogate virus neutralization test (sVNT) (Genscript catalog number L00847-A) (5) and conventional virus neutralization tests (cVNT) (5) (Fig. 1A). Further details are provided in Text S1.

10.1128/mbio.01423-22.1 TEXT S1 This file provides further details of the supplementary materials and the phase III clinical trial methods reported in the manuscript, along with related references. Download Text S1, DOCX file, 0.04 MB. Copyright © 2022 Schultz et al. 2022 Schultz et al. https://creativecommons.org/licenses/by/4.0/ This content is distributed under the terms of the Creative Commons Attribution 4.0 International license.

A pseudotyped virus neutralization test (pVNT) assay was performed to assess the capacity of the antibodies against SARS-CoV-2 VOC in 30 volunteers of the group of 77 previously analyzed by sVNT, as previously reported (10, 13). Also, cVNT was performed on 19 volunteers to evaluate the capacity of the antibodies against the SARS-CoV-2 Delta variant. Further details are provided in Text S1.

The number of SFC for IFN-γ were determined by ELISPOT, and the expression of activation-induced markers (AIM) by T cells was evaluated by flow cytometry in 40 volunteers of the 77 previously analyzed (Fig. 1A), after stimulating PBMCs with four megapools (MPs) of peptides derived from the proteome of SARS-CoV-2 (35): MP-S, MP-R, MP-CD8-A, and MP-CD8-B (35). Samples from 30 of the previously analyzed volunteers were also stimulated with three MPs of VOCs, provided by La Jolla Institute for Immunology (10), to evaluate T cell activation 4 weeks after the booster dose. Assays were performed according to the manufacturer’s instructions and as reported previously (5). Further details for the ELISPOT assay, antibodies used for flow cytometry, and the respective protocols can be found in the Text S1 and Table S3.

10.1128/mbio.01423-22.8 TABLE S3 Reagents used in flow cytometry. Download Table S3, DOCX file, 0.02 MB. Copyright © 2022 Schultz et al. 2022 Schultz et al. https://creativecommons.org/licenses/by/4.0/ This content is distributed under the terms of the Creative Commons Attribution 4.0 International license.

IL-2 and IFN-γ secretion were evaluated in the supernatants of 22 volunteers previously stimulated for 20 h with a SARS-CoV-2 MP of peptides derived from the Spike protein of VOCs, using a Luminex 200 xMap multiplex system (Luminex Corporation, Austin, TX). The limit of detection for the cytokines measured ranged from 4.2 to 13,390 pg/mL, according to the manufacturer's instructions. Further experimental details can be found in Text S1.

### Statistical analyses.

Statistical differences for the immunogenicity results were evaluated with a repeated-measures analysis of variance (ANOVA) with the Geisser-Greenhouse correction and Dunnet’s *a posteriori* multiple-comparisons test to compare the booster dose and the other visits. Analyses were performed over 10 logs of the data for neutralizing antibodies by sVNT, cVNT, and pVNT. Cellular immune responses were analyzed by a Friedman test for repeated measures for ELISPOT and flow cytometry data to compare responses to the booster dose and the other visits. Secretions of cytokines were compared between the secretion induced by the ancestral strain against the VOCs Delta and Omicron by a repeated-measures ANOVA. The significance level was set at 0.05 for all the analyses. All data were analyzed with GraphPad Prism 9.0.1.

## References

[1] Mallapaty S. 2021. WHO approval of Chinese CoronaVac COVID vaccine will be crucial to curbing pandemic. Nature 594:161–162. doi:10.1038/d41586-021-01497-8. 34089030

[2] Gao Q, Bao L, Mao H, Wang L, Xu K, Yang M, Li Y, Zhu L, Wang N, Lv Z, Gao H, Ge X, Kan B, Hu Y, Liu J, Cai F, Jiang D, Yin Y, Qin C, Li J, Gong X, Lou X, Shi W, Wu D, Zhang H, Zhu L, Deng W, Li Y, Lu J, Li C, Wang X, Yin W, Zhang Y, Qin C. 2020. Development of an inactivated vaccine candidate for SARS-CoV-2. Science 369:77–81. doi:10.1126/science.abc1932. 32376603 PMC7202686

[3] Wu Z, Hu Y, Xu M, Chen Z, Yang W, Jiang Z, Li M, Jin H, Cui G, Chen P, Wang L, Zhao G, Ding Y, Zhao Y, Yin W. 2021. Safety, tolerability, and immunogenicity of an inactivated SARS-CoV-2 vaccine (CoronaVac) in healthy adults aged 60 years and older: a randomised, double-blind, placebo-controlled, phase 1/2 clinical trial. Lancet Infect Dis 21:803–812. doi:10.1016/S1473-3099(20)30987-7. 33548194 PMC7906628

[4] Zhang Y, Zeng G, Pan H, Li C, Hu Y, Chu K, Han W, Chen Z, Tang R, Yin W, Chen X, Hu Y, Liu X, Jiang C, Li J, Yang M, Song Y, Wang X, Gao Q, Zhu F. 2021. Safety, tolerability, and immunogenicity of an inactivated SARS-CoV-2 vaccine in healthy adults aged 18–59 years: a randomised, double-blind, placebo-controlled, phase 1/2 clinical trial. Lancet Infect Dis 21:181–192. doi:10.1016/S1473-3099(20)30843-4. 33217362 PMC7832443

[5] Bueno SM, Abarca K, González PA, Gálvez NMS, Soto JA, Duarte LF, Schultz BM, Pacheco GA, González LA, Vázquez Y, Ríos M, Melo-González F, Rivera-Pérez D, Iturriaga C, Urzúa M, Domínguez A, Andrade CA, Berrios RV, Canedo-Marroquín G, Covián C, Moreno-Tapia D, Saavedra F, Vallejos OP, Donato P, Espinoza P, Fuentes D, González M, Guzmán P, Muñoz-Venturelli P, Pérez CM, Potin M, Rojas Á, Fasce R, Fernández J, Mora J, Ramírez E, Gaete-Argel A, Oyarzún-Arrau A, Valiente-Echeverría F, Soto-Rifo R, Weiskopf D, Sette A, Zeng G, Meng W, González-Aramundiz JV, Kalergis AM, CoronaVac03CL Study Group. 2021. Safety and Immunogenicity of an Inactivated SARS-CoV-2 Vaccine in a Subgroup of Healthy Adults in Chile. Clin Infect Dis ciab823. doi:10.1093/cid/ciab823. PMC9402626 34537835

[6] Duarte LF, Gálvez NMS, Iturriaga C, Melo-González F, Soto JA, Schultz BM, Urzúa M, González LA, Vázquez Y, Ríos M, Berríos-Rojas RV, Rivera-Pérez D, Moreno-Tapia D, Pacheco GA, Vallejos OP, Hoppe-Elsholz G, Navarrete MS, Rojas Á, Fasce RA, Fernández J, Mora J, Ramírez E, Zeng G, Meng W, González-Aramundiz JV, González PA, Abarca K, Bueno SM, Kalergis AM. 2021. Immune profile and clinical outcome of breakthrough cases after vaccination with an inactivated SARS-CoV-2 vaccine. Front Immunol 12:3884. doi:10.3389/fimmu.2021.742914. PMC8511644 34659237

[7] Jara A, Undurraga EA, González C, Paredes F, Fontecilla T, Jara G, Pizarro A, Acevedo J, Leo K, Leon F, Sans C, Leighton P, Suárez P, García-Escorza H, Araos R. 2021. Effectiveness of an inactivated SARS-CoV-2 vaccine in Chile. N Engl J Med 385:875–884. doi:10.1056/NEJMoa2107715. 34233097 PMC8279092

[8] Khoury DS, Cromer D, Reynaldi A, Schlub TE, Wheatley AK, Juno JA, Subbarao K, Kent SJ, Triccas JA, Davenport MP. 2021. Neutralizing antibody levels are highly predictive of immune protection from symptomatic SARS-CoV-2 infection. Nat Med 27:1205–1211. doi:10.1038/s41591-021-01377-8. 34002089

[9] Sauré D, O'Ryan M, Torres JP, Zuniga M, Santelices E, Basso LJ. 2022. Dynamic IgG seropositivity after rollout of CoronaVac and BNT162b2 COVID-19 vaccines in Chile: a sentinel surveillance study. Lancet Infect Dis 22:56–63. doi:10.1016/S1473-3099(21)00479-5. 34509185 PMC8428469

[10] Melo-González F, Soto JA, González LA, Fernández J, Duarte LF, Schultz BM, Gálvez NMS, Pacheco GA, Ríos M, Vázquez Y, Rivera-Pérez D, Moreno-Tapia D, Iturriaga C, Vallejos OP, Berríos-Rojas RV, Hoppe-Elsholz G, Urzúa M, Bruneau N, Fasce RA, Mora J, Grifoni A, Sette A, Weiskopf D, Zeng G, Meng W, González-Aramundiz JV, González PA, Abarca K, Ramírez E, Kalergis AM, Bueno SM. 2021. Recognition of variants of concern by antibodies and T cells induced by a SARS-CoV-2 inactivated vaccine. Front Immunol 12. doi:10.3389/fimmu.2021.747830. PMC8630786 34858404

[11] Clemens SAC, Weckx L, Clemens R, Mendes AVA, Souza AR, Silveira MV, da Guarda SNF, de Nobrega MM, Pinto MI, de M, Gonzalez IGS, Salvador N, Franco MM, Mendonça RN, de A, Oliveira ISQ, Souza BS, de F, Fraga M, Aley P, Bibi S, Cantrell L, Dejnirattisai W, Liu X, Mongkolsapaya J, Supasa P, Screaton GR, Lambe T, Voysey M, Pollard AJ, RHH-001 Study Team. 2022. Heterologous versus homologous COVID-19 booster vaccination in previous recipients of two doses of CoronaVac COVID-19 vaccine in Brazil (RHH-001): a phase 4, non-inferiority, single blind, randomised study. Lancet 399:521–529. doi:10.1016/S0140-6736(22)00094-0. 35074136 PMC8782575

[12] Pérez-Then E, Lucas C, Monteiro VS, Miric M, Brache V, Cochon L, Vogels CBF, Malik AA, De la Cruz E, Jorge A, De los Santos M, Leon P, Breban MI, Billig K, Yildirim I, Pearson C, Downing R, Gagnon E, Muyombwe A, Razeq J, Campbell M, Ko AI, Omer SB, Grubaugh ND, Vermund SH, Iwasaki A. 2022. Neutralizing antibodies against the SARS-CoV-2 Delta and Omicron variants following heterologous CoronaVac plus BNT162b2 booster vaccination. Nat Med 28:481–481. doi:10.1038/s41591-022-01705-6. 35051990 PMC8938264

[13] Acevedo ML, Gaete-Argel A, Alonso-Palomares L, de Oca MM, Bustamante A, Gaggero A, Paredes F, Cortes CP, Pantano S, Martínez-Valdebenito C, Angulo J, Le Corre N, Ferrés M, Navarrete MA, Valiente-Echeverría F, Soto-Rifo R. 2022. Differential neutralizing antibody responses elicited by CoronaVac and BNT162b2 against SARS-CoV-2 Lambda in Chile. Nat Microbiol 7:524–529. doi:10.1038/s41564-022-01092-1. 35365787

[14] Pan H, Wu Q, Zeng G, Yang J, Jiang D, Deng X, Chu K, Zheng W, Zhu F, Yu H, Yin W. 2021. Immunogenicity and safety of a third dose, and immune persistence of CoronaVac vaccine in healthy adults aged 18–59 years: interim results from a double-blind, randomized, placebo-controlled phase 2 clinical trial. medRxiv 2021.07.23.21261026.

[15] Abarca K, Iturriaga C, Urzúa M, Le Corre N, Pineda A, Fernández C, Domínguez A, González PA, Bueno SM, Donato P, Espinoza P, Fuentes D, González M, Guzmán P, Muñoz-Venturelli P, Pérez CM, Potin M, Rojas Á, González-Aramundiz JV, Gálvez NMS, Aguirre-Boza F, Aljaro S, Bátiz LF, Campisto Y, Cepeda M, Cortés A, López S, Pérez ML, Schilling A, Kalergis AM, on behalf of the CoronaVac03CL Study Group. 2022. Safety and Non-Inferiority Evaluation of Two Immunization Schedules with an Inactivated SARS-CoV-2 Vaccine in Adults: A Randomized Clinical Trial. Vaccines 10:1082. doi:10.3390/vaccines10071082. 35891246 PMC9323976

[16] Lumley SF, O'Donnell D, Stoesser NE, Matthews PC, Howarth A, Hatch SB, Marsden BD, Cox S, James T, Warren F, Peck LJ, Ritter TG, de Toledo Z, Warren L, Axten D, Cornall RJ, Jones EY, Stuart DI, Screaton G, Ebner D, Hoosdally S, Chand M, Crook DW, O'Donnell A-M, Conlon CP, Pouwels KB, Walker AS, Peto TEA, Hopkins S, Walker TM, Jeffery K, Eyre DW, Oxford University Hospitals Staff Testing Group. 2021. Antibody status and incidence of SARS-CoV-2 infection in health care workers. N Engl J Med 384:533–540. doi:10.1056/NEJMoa2034545. 33369366 PMC7781098

[17] Sabino EC, Buss LF, Carvalho MPS, Prete CA, Crispim MAE, Fraiji NA, Pereira RHM, Parag KV, da Silva Peixoto P, Kraemer MUG, Oikawa MK, Salomon T, Cucunuba ZM, Castro MC, de Souza Santos AA, Nascimento VH, Pereira HS, Ferguson NM, Pybus OG, Kucharski A, Busch MP, Dye C, Faria NR. 2021. Resurgence of COVID-19 in Manaus, Brazil, despite high seroprevalence. Lancet 397:452–455. doi:10.1016/S0140-6736(21)00183-5. 33515491 PMC7906746

[18] Wang K, Cao Y, Zhou Y, Wu J, Jia Z, Hu Y, Yisimayi A, Fu W, Wang L, Liu P, Fan K, Chen R, Wang L, Li J, Wang Y, Ge X, Zhang Q, Wu J, Wang N, Wu W, Gao Y, Miao J, Jiang Y, Qin L, Zhu L, Huang W, Zhang Y, Zhang H, Li B, Gao Q, Xie XS, Wang Y, Wang Q, Wang X. 2021. A third dose of inactivated vaccine augments the potency, breadth, and duration of anamnestic responses against SARS-CoV-2. medRxiv 2021.09.02.21261735.10.1093/procel/pwae03338801319

[19] Bar-On YM, Goldberg Y, Mandel M, Bodenheimer O, Freedman L, Kalkstein N, Mizrahi B, Alroy-Preis S, Ash N, Milo R, Huppert A. 2021. Protection of BNT162b2 vaccine booster against Covid-19 in Israel. N Engl J Med 385:1393–1400. doi:10.1056/NEJMoa2114255. 34525275 PMC8461568

[20] Ministerio de Salud, Chile. 2021. Immunization campaign against SARS-CoV-2: early estimates of the effectiveness of booster shots in Chile. https://www.minsal.cl/wp-content/uploads/2021/10/2021-10-07-EFECTIVIDAD-DOSIS-DE-REFUERZO_ENG.pdf. Accessed 10 December 2021.

[21] Li M, Yang J, Wang L, Wu Q, Wu Z, Zheng W, Wang L, Lu W, Deng X, Peng C, Han B, Zhao Y, Yu H, Yin W. 2021. A booster dose is immunogenic and will be needed for older adults who have completed two doses vaccination with CoronaVac: a randomised, double-blind, placebo-controlled, phase 1/2 clinical trial. medRxiv 2021.08.03.21261544.

[22] Mateus J, Dan JM, Zhang Z, Rydyznski Moderbacher C, Lammers M, Goodwin B, Sette A, Crotty S, Weiskopf D. 2021. Low-dose mRNA-1273 COVID-19 vaccine generates durable memory enhanced by cross-reactive T cells. Science 374 doi:10.1126/science.abj9853. PMC8542617 34519540

[23] Guerrera G, Picozza M, D’Orso S, Placido R, Pirronello M, Verdiani A, Termine A, Fabrizio C, Giannessi F, Sambucci M, Balice MP, Caltagirone C, Salvia A, Rossini A, Battistini L, Borsellino G. 2021. BNT162b2 vaccination induces durable SARS-CoV-2 specific T cells with a stem cell memory phenotype. Sci Immunol 6. doi:10.1126/sciimmunol.abl5344. 34726470

[24] Mok CKP, Cohen CA, Cheng SMS, Chen C, Kwok K-O, Yiu K, Chan T-O, Bull M, Ling KC, Dai Z, Ng SS, Lui GC-Y, Wu C, Amarasinghe GK, Leung DW, Wong SYS, Valkenburg SA, Peiris M, Hui DS. 2022. Comparison of the immunogenicity of BNT162b2 and CoronaVac COVID-19 vaccines in Hong Kong. Respirology 27:301–310. doi:10.1111/resp.14191. 34820940 PMC8934254

[25] Escobar A, Reyes-López FE, Acevedo ML, Alonso-Palomares L, Valiente-Echeverría F, Soto-Rifo R, Portillo H, Gatica J, Flores I, Nova-Lamperti E, Barrera-Avalos C, Bono MR, Vargas L, Simon V, Leiva-Salcedo E, Vial C, Hormazabal J, Cortes LJ, Valdés D, Sandino AM, Imarai M, Acuña-Castillo C. 2022. Evaluation of the immune response induced by CoronaVac 28-day schedule vaccination in a healthy population group. Front Immunol 12. doi:10.3389/fimmu.2021.766278. PMC8841433 35173705

[26] Atmar RL, Lyke KE, Deming ME, Jackson LA, Branche AR, el Sahly HM, Rostad CA, Martin JM, Johnston C, Rupp RE, Mulligan MJ, Brady RC, Frenck RW, Bäcker M, Kottkamp AC, Babu TM, Rajakumar K, Edupuganti S, Dobrzynski D, Coler RN, Posavad CM, Archer JI, Crandon S, Nayak SU, Szydlo D, Zemanek JA, Dominguez Islas CP, Brown ER, Suthar MS, McElrath MJ, McDermott AB, O’Connell SE, Montefiori DC, Eaton A, Neuzil KM, Stephens DS, Roberts PC, Beigel JH. 2022. Homologous and heterologous Covid-19 booster vaccinations. N Engl J Med 386:1046–1057. doi:10.1056/NEJMoa2116414. 35081293 PMC8820244

[27] Cao Y, Hao X, Wang X, Wu Q, Song R, Zhao D, Song W, Wang Y, Yisimayi A, Wang W, Zhang W, Du J, Yu H, Xie XS, Jin R. 2022. Humoral immunogenicity and reactogenicity of CoronaVac or ZF2001 booster after two doses of inactivated vaccine. Cell Res 32:107–109. doi:10.1038/s41422-021-00596-5. 34862467 PMC8640508

[28] Viana R, Moyo S, Amoako DG, Tegally H, Scheepers C, Althaus CL, Anyaneji UJ, Bester PA, Boni MF, Chand M, Choga WT, Colquhoun R, Davids M, Deforche K, Doolabh D, Du Plessis L, Engelbrecht S, Everatt J, Giandhari J, Giovanetti M, Hardie D, Hill V, Hsiao N-Y, Iranzadeh A, Ismail A, Joseph C, Joseph R, Koopile L, Kosakovsky Pond SL, Kraemer MUG, Kuate-Lere L, Laguda-Akingba O, Lesetedi-Mafoko O, Lessells RJ, Lockman S, Lucaci AG, Maharaj A, Mahlangu B, Maponga T, Mahlakwane K, Makatini Z, Marais G, Maruapula D, Masupu K, Matshaba M, Mayaphi S, Mbhele N, Mbulawa MB, Mendes A, Mlisana K, et al. 2022. Rapid epidemic expansion of the SARS-CoV-2 Omicron variant in southern Africa. Nature doi:10.1038/d41586-021-03832-5. PMC8942855 35042229

[29] Ai J, Zhang H, Zhang Q, Zhang Y, Lin K, Fu Z, Song J, Zhao Y, Fan M, Wang H, Qiu C, Zhou Y, Zhang W. 2022. Recombinant protein subunit vaccine booster following two-dose inactivated vaccines dramatically enhanced anti-RBD responses and neutralizing titers against SARS-CoV-2 and variants of concern. Cell Res 32:103–106. doi:10.1038/s41422-021-00590-x. 34815511 PMC8609258

[30] Tarke A, Sidney J, Methot N, Yu ED, Zhang Y, Dan JM, Goodwin B, Rubiro P, Sutherland A, Wang E, Frazier A, Ramirez SI, Rawlings SA, Smith DM, da Silva Antunes R, Peters B, Scheuermann RH, Weiskopf D, Crotty S, Grifoni A, Sette A. 2021. Impact of SARS-CoV-2 variants on the total CD4+ and CD8+ T cell reactivity in infected or vaccinated individuals. Cell Rep Med 2:100355. doi:10.1016/j.xcrm.2021.100355. 34230917 PMC8249675

[31] Gao Y, Cai C, Grifoni A, Müller TR, Niessl J, Olofsson A, Humbert M, Hansson L, Österborg A, Bergman P, Chen P, Olsson A, Sandberg JK, Weiskopf D, Price DA, Ljunggren H-G, Karlsson AC, Sette A, Aleman S, Buggert M. 2022. Ancestral SARS-CoV-2-specific T cells cross-recognize the Omicron variant. Nat Med 2022 doi:10.1038/d41591-022-00017-z. PMC8938268 35042228

[32] GeurtsvanKessel CH, Geers D, Schmitz KS, Mykytyn AZ, Lamers MM, Bogers S, Scherbeijn S, Gommers L, Sablerolles RSG, Nieuwkoop NN, Rijsbergen LC, van Dijk LLA, de Wilde J, Alblas K, Breugem TI, Rijnders BJA, de Jager H, Weiskopf D, van der Kuy PHM, Sette A, Koopmans MPG, Grifoni A, Haagmans BL, de Vries RD. 2022. Divergent SARS-CoV-2 Omicron–reactive T and B cell responses in COVID-19 vaccine recipients. Sci Immunol 7:eabo2202. doi:10.1126/sciimmunol.abo2202. 35113647 PMC8939771

[33] Tarke A, Coelho CH, Zhang Z, Dan JM, Yu ED, Methot N, Bloom NI, Goodwin B, Phillips E, Mallal S, Sidney J, Filaci G, Weiskopf D, da Silva Antunes R, Crotty S, Grifoni A, Sette A. 2022. SARS-CoV-2 vaccination induces immunological T cell memory able to cross-recognize variants from Alpha to Omicron. Cell 185:847–859.e11. doi:10.1016/j.cell.2022.01.015. 35139340 PMC8784649

[34] World medical association. 2013. World Medical Association declaration of Helsinki: ethical principles for medical research involving human subjects. JAMA 310:2191–2194. doi:10.1001/jama.2013.281053. 24141714

[35] Grifoni A, Weiskopf D, Ramirez SI, Mateus J, Dan JM, Moderbacher CR, Rawlings SA, Sutherland A, Premkumar L, Jadi RS, Marrama D, de Silva AM, Frazier A, Carlin AF, Greenbaum JA, Peters B, Krammer F, Smith DM, Crotty S, Sette A. 2020. Targets of T cell responses to SARS-CoV-2 coronavirus in humans with COVID-19 disease and unexposed individuals. Cell 181:1489–1501.e15. doi:10.1016/j.cell.2020.05.015. 32473127 PMC7237901

