# Supplementary material for: A Booster Dose of CoronaVac Increases Neutralizing Antibodies and T Cells that Recognize Delta and Omicron Variants of Concern
Source: mBio. 2022 Aug 10;13(4):e01423-22. doi: 10.1128/mbio.01423-22 (PMC9426482; doi:10.1128/mbio.01423-22)
Supplement: TABLE S1 [file mbio.01423-22-s0006.docx]

**Supplementary Table 1.** Solicited local adverse events after inoculation in volunteers classified by arm age group after the booster dose.

| **Local adverse Reactions** | **Booster dose**  **(n=1440)** |
| --- | --- |
| **1. Pain (%)** | 462 (32,1) |
| <60 years | 396 (38,9) |
| ≥60 years | 66 (15,6) |
| *p-value (a)* | ***<0,001*** |
| **2. Induration (%)** | 64 (4,4) |
| <60 years | 57 (5,6) |
| ≥60 years | 7 (1,7) |
| *p-value (a)* | ***0,001*** |
| **3. Pruritus (%)** | 40 (2,8) |
| <60 years | 35 (3,4) |
| ≥60 years | 5 (1,2) |
| *p-value (a)* | *0,018* |
| **4. Erythema (%)** | 34 (2,4) |
| <60 years | 32 (3,1) |
| ≥60 years | 2 (0,5) |
| *p-value (a)* | ***0,002*** |
| **5. Swelling (%)** | 66 (4,6) |
| <60 years | 62 (6,1) |
| ≥60 years | 4 (0,9) |
| *p-value (a)* | ***<0,001*** |
