# Supplementary material for: A Booster Dose of CoronaVac Increases Neutralizing Antibodies and T Cells that Recognize Delta and Omicron Variants of Concern
Source: mBio. 2022 Aug 10;13(4):e01423-22. doi: 10.1128/mbio.01423-22 (PMC9426482; doi:10.1128/mbio.01423-22)
Supplement: TABLE S2 [file mbio.01423-22-s0007.docx]

**Supplementary Table 2.** Solicited systemic adverse events after inoculation in volunteers classified by arm age group after the booster dose.

| **Systemic adverse Reactions** | **Booster doce (n=1440**) |
| --- | --- |
| **1.Headache** | 284 (19,7) |
| <60 years | 223 (21,9) |
| ≥60 years | 61 (14,5) |
| *p-value (a)* | ***0,001*** |
| 2. Fatigue | 205 (14,2) |
| <60 years | 159 (15,6) |
| ≥60 years | 46 (10,9) |
| *p-value (a)* | *0,020* |
| **3. Muscle pain** | 216 (15,0) |
| <60 years | 171 (16,8) |
| ≥60 years | 45 (10,7) |
| *p-value (a)* | ***0,003*** |
| **4. Diarrhea** | 84 (5,8) |
| <60 years | 66 (6,5) |
| ≥60 years | 18 (4,3) |
| *p-value (a)* | *0,102* |
| **5. Nausea** | 63 (4,4) |
| <60 years | 51 (5,0) |
| ≥60 years | 12 (2,8) |
| *p-value (a)* | *0,067* |
| **6. Anorexy** | 52 (3,6) |
| <60 years | 45 (4,4) |
| ≥60 years | 7 (1,7) |
| *p-value (a)* | *0,011* |
| **7. Atralgia** | 101 (7,0) |
| <60 years | 74 (7,3) |
| ≥60 years | 27 (6,4) |
| *p-value (a)* | *0,556* |
| **8. Overall itching** | 19 (1,3) |
| <60 years | 16 (1,6) |
| ≥60 years | 3 (0,7) |
| *p-value (a)* | *0,193* |
| **9. Exanthema** | 11 (0,8) |
| <60 years | 9 (0,9) |
| ≥60 years | 2 (0,5) |
| *p-value (a)* | *0,524* |
| **10. Allergic reaction** | 15 (1,0) |
| <60 years | 12 (1,2) |
| ≥60 years | 3 (0,7) |
| *p-value (a)* | *0,574* |
| **11. Vomiting** | 6 (0,4) |
| <60 years | 6 (0,6) |
| ≥60 years | 0 (0,0) |
| *p-value (a)* | *0,189* |
| **12. Fever (>37.8ºC)** | 3 (0,2) |
| <60 years | 3 (0,3) |
| ≥60 years | 0 (0,0) |
| *p-value (a)* | *0,560* |
