# Supplementary figures and images for: A Booster Dose of CoronaVac Increases Neutralizing Antibodies and T Cells that Recognize Delta and Omicron Variants of Concern
Source: mBio. 2022 Aug 10;13(4):e01423-22. doi: 10.1128/mbio.01423-22 (PMC9426482; doi:10.1128/mbio.01423-22)

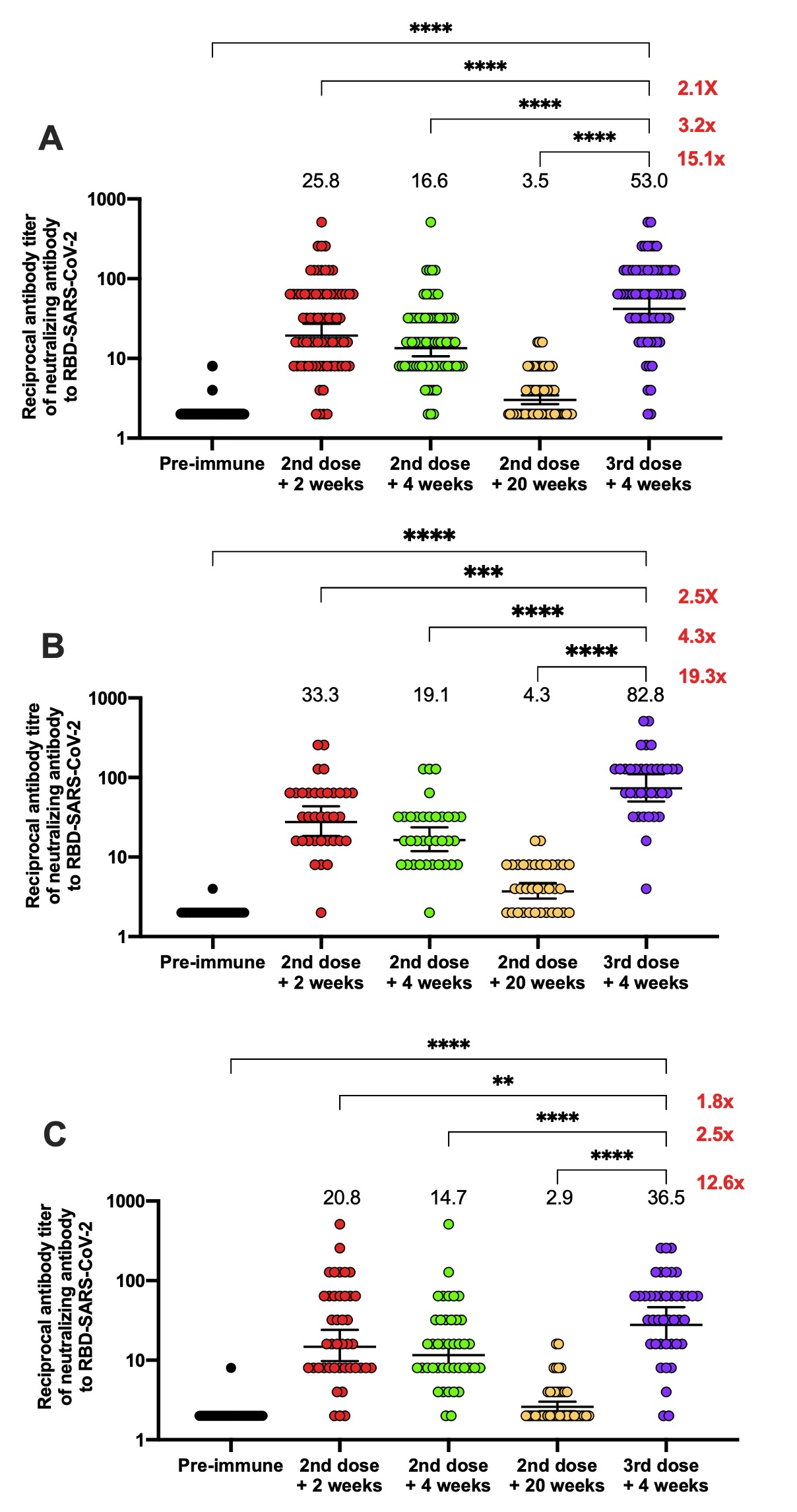

Supplement: FIG S1 [file mbio.01423-22-s0002.tif]

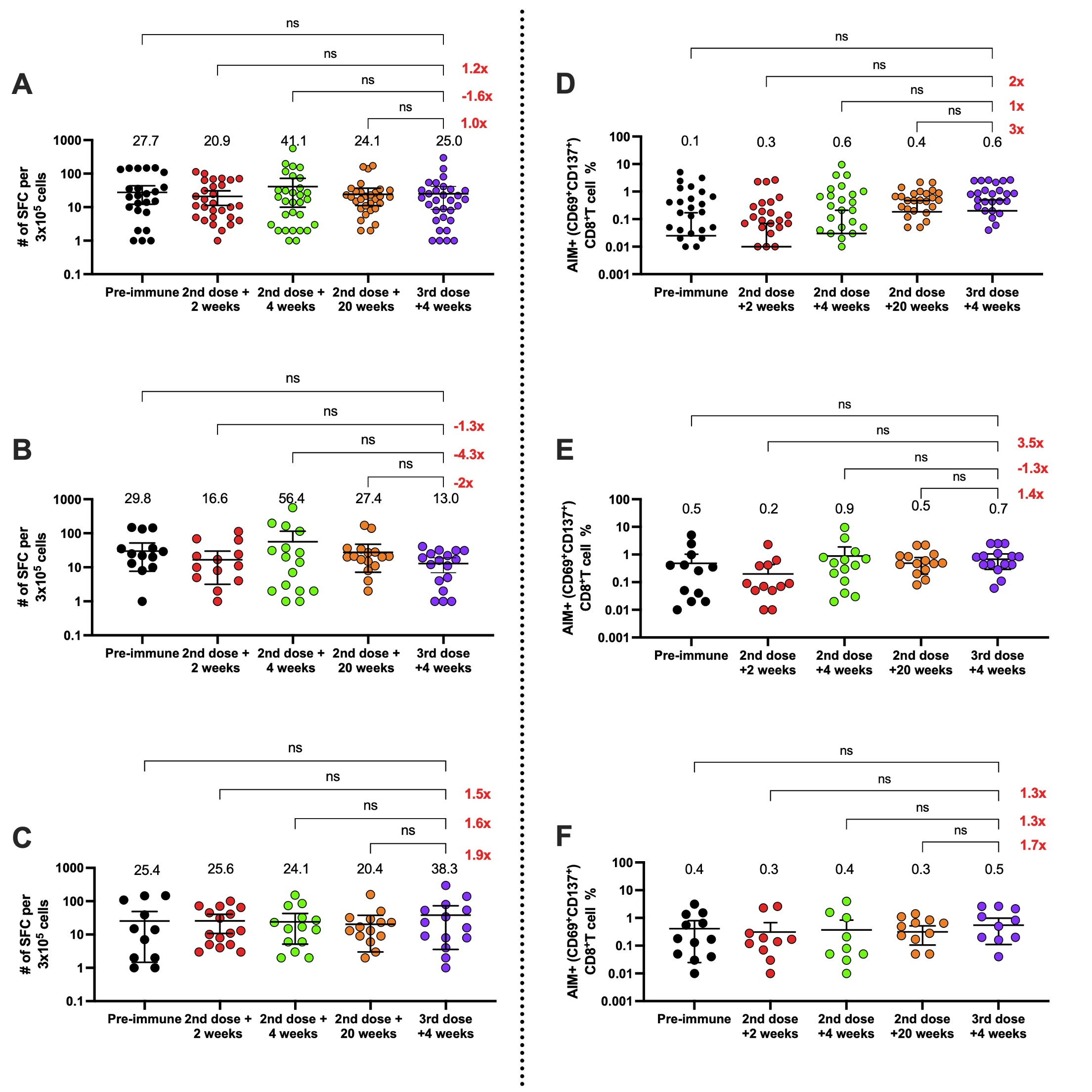

Supplement: FIG S2 [file mbio.01423-22-s0003.tif]

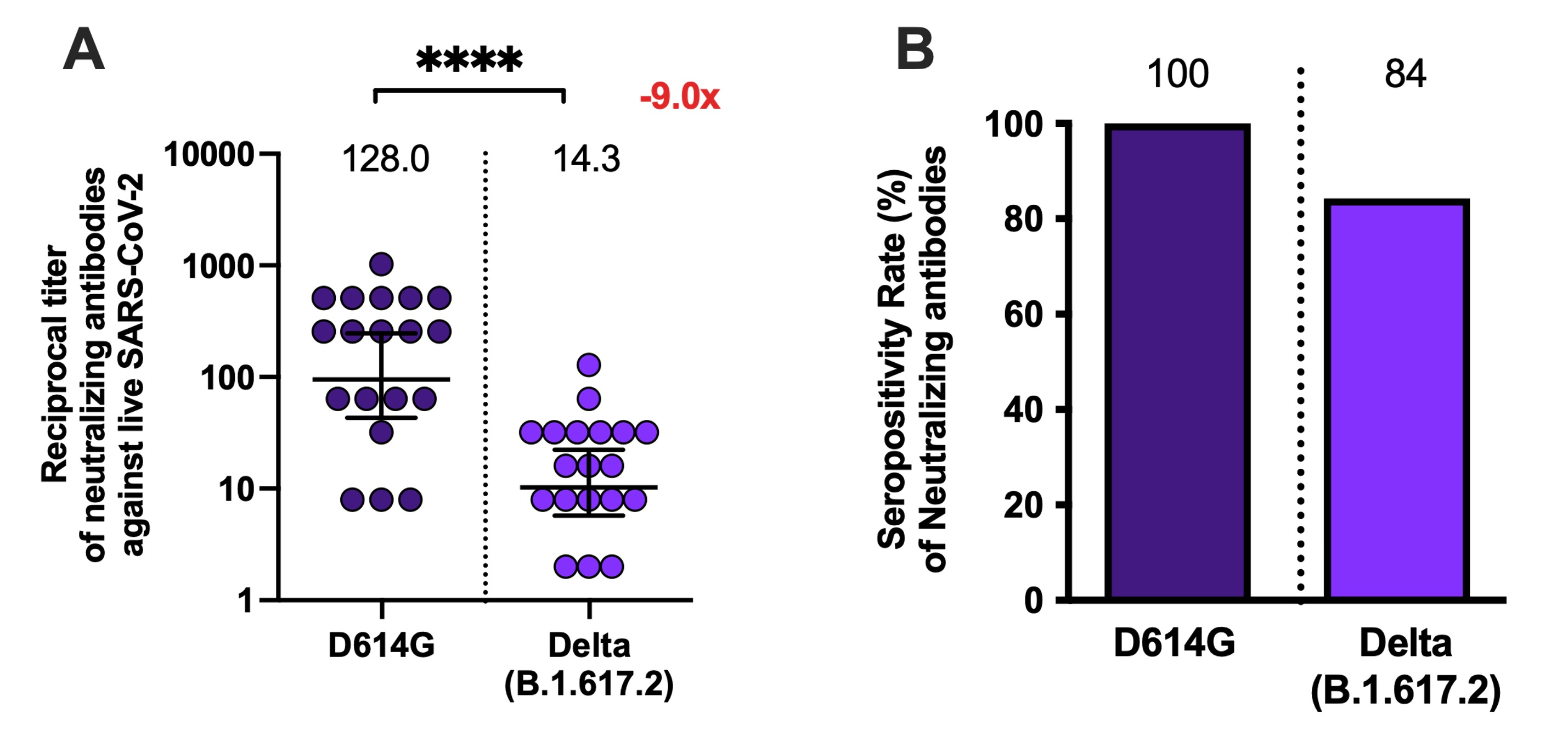

Supplement: FIG S3 [file mbio.01423-22-s0004.tif]

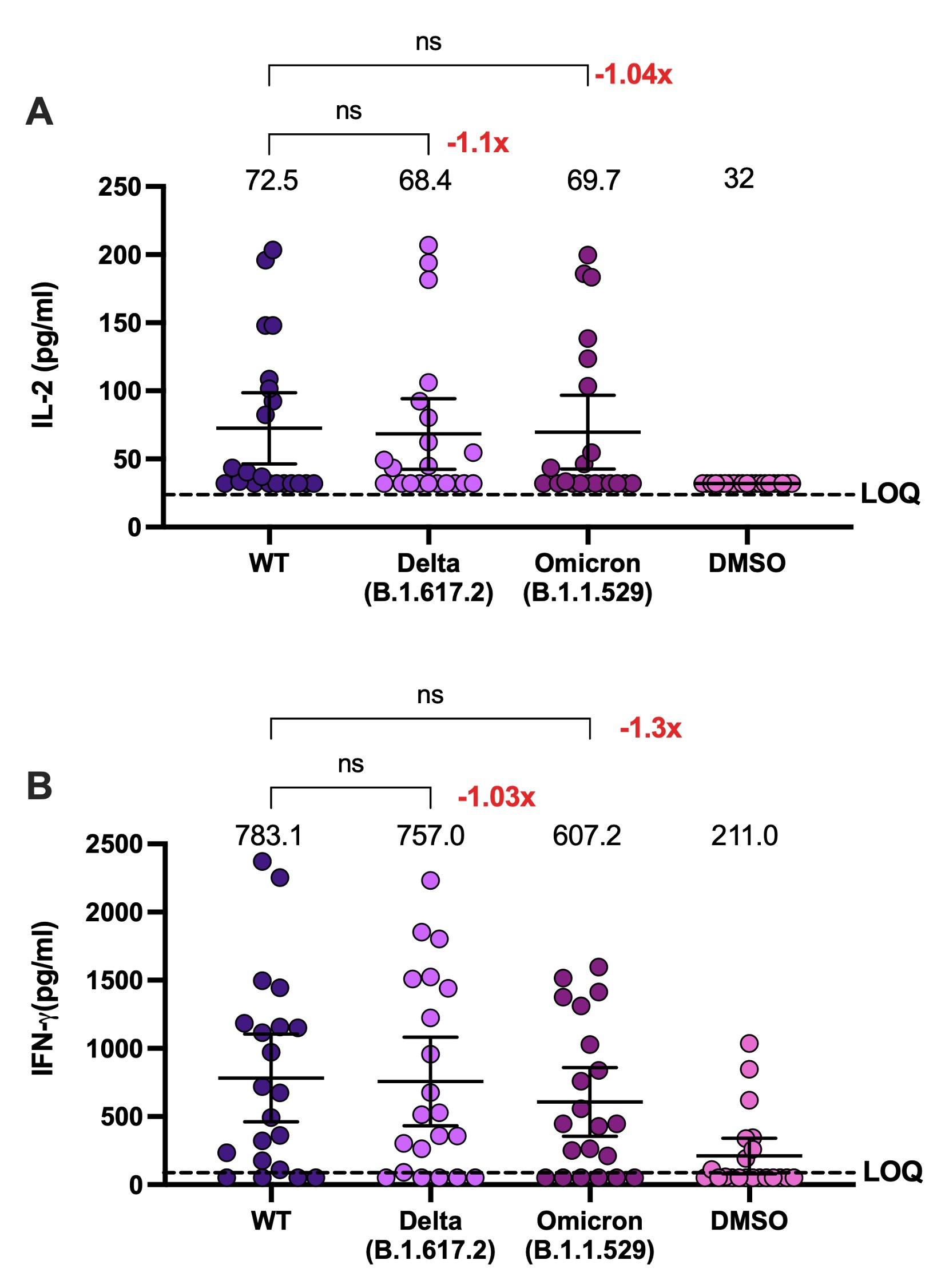

Supplement: FIG S4 [file mbio.01423-22-s0005.tif]
