# Supplementary material for: A Booster Dose of CoronaVac Increases Neutralizing Antibodies and T Cells that Recognize Delta and Omicron Variants of Concern
Source: mBio. 2022 Aug 10;13(4):e01423-22. doi: 10.1128/mbio.01423-22 (PMC9426482; doi:10.1128/mbio.01423-22)
Supplement: TABLE S4 [file mbio.01423-22-s0009.docx]

**Supplementary Table 4: Seropositivity rates, Geometric Mean Titer (GMT) of circulating neutralizing antibodies against SARS-CoV-2 RBD of D614G and Delta variant.**

|  | **Variant** | **D614G** | **Delta (B1.617.2)** |
| --- | --- | --- | --- |
| cVNT | **Indicators** | **3rd dose + 4 weeks** | **3rd dose + 4 weeks** |
|  | **Seropositivity n/N** | **19/19** | **16/19** |
|  | **(%)** | **100** | **84** |
|  | **GMT** | **128.0** | **14.3** |
|  | **95% CI** | **60.6-270.2** | **8.2-25.1** |

*GMT: Geometric mean titer.*
