# Supplementary material for: A Booster Dose of CoronaVac Increases Neutralizing Antibodies and T Cells that Recognize Delta and Omicron Variants of Concern
Source: mBio. 2022 Aug 10;13(4):e01423-22. doi: 10.1128/mbio.01423-22 (PMC9426482; doi:10.1128/mbio.01423-22)
Supplement: TEXT S1 [file mbio.01423-22-s0001.docx]

**Supplementary information: Material and methods**.

**Study design, volunteers, and vaccine**

The clinical trial (clinicaltrials.gov NCT04651790) was conducted in Chile at eight different sites, six located in Santiago city (Metropolitan Region) and two in the V Region of Valparaiso. The committee members that reviewed and approved this trial protocol (Institutional Scientific Ethical Committee of Health Sciences, Pontificia Universidad Católica de Chile, Approval #200708006) were: Claudia Uribe PUC/Committee president; Colomba Cofré/Committee Vice-president; Andréa Villagrán/Executive secretary; Jorge Muñoz/External Lawyer; Gustavo Kaltwasser/External member; Alysa Garay/Community representative; Marisa Torres/Public Health Department; Carolina Méndez/Speech therapy representative; Luis Villarroel/Public Health Department; Pablo Brockman/Respiratory Diseases in Children Department. Website: http://eticayseguridad.uc.cl/comite-etico-cientifico-facultad-de-medicina-uc.html).   Informed consent was obtained from all volunteers upon enrollment. Volunteers receive two doses of CoronaVac^®^ (3 µg or 600SU of inactivated SARS-CoV-2 inactivated along with alum adjuvant) in a four-week interval (0-28-day immunization schedule) and then a booster dose five months after the second dose.

**Procedures**

For the isolation of sera, 20 mL of blood were collected in anticoagulant tubes and distributed in 2 tubes of 10 mL per volunteer (BD Vacutainer Clot Activator tubes #367896). Blood was allowed to clot for at least 1h at RT. Samples were then centrifuged in a refrigerated centrifuge with a horizontal rotor at 1,300 x *g* for 10 min at 22ºC. Serum was collected and stored at –80°C until use. Hemolyzed samples were rejected. For the isolation of PBMCs, blood was collected in 3 heparinized tubes (BD Vacutainer #367874, 10 mL) and stored at RT until processing. Samples were diluted with PBS (1:1) and centrifuged for 10 min at 1,200 x g (RT) in SepMate™ tubes (StemCell Technologies) with density-gradient medium (Lymphoprep). The plasma was then and PBMCs were isolated by pouring them into a clean tube. Isolated PBMCs were washed twice with sterile PBS, counted, and cryopreserved in FBS (Industrial Biologicals) and 10% DMSO (Chem Cruz). All PBMC samples were stored in liquid nitrogen until use.

The neutralizing capacities of the antibodies in the samples of the volunteers were evaluated through the SARS-CoV-2 sVNT kit from Genscript (cat. number L00847-A). Assays were performed according to the instructions of the manufacturer. Briefly, serial dilutions of the serum were prepared and then incubated with the HRP-RBD reagent supplied in the kit for 30 min at 37ºC to allow the binding of neutralizing antibodies to S1-RBD. Sera and controls previously incubated with the HRP-RBD were added to the ELISA plate pre-coated with the human angiotensin-converting enzyme 2 (hACE2) protein and incubated for 15 min at 37ºC. After the incubation, samples were discarded, and plates were washed. Finally, a developing solution provided in the kit was added for 15 min at RT and then quenched with a stop solution also supplied. Plates were read at 450 nm in a microplate reader (Biotek, Ref. 1506021). The inhibition rate of HRP-RBD binding was calculated as follows: 100 * [OD450nm value of negative control - OD450nm value of sample] / [OD450nm of negative control]. Samples with a percentage of inhibition ≤30 at the lowest dilution (1:4) were assigned as seronegative with a titer of 2. A sample was considered seropositive when its titer is higher than the pre-immune titer. The methods used in this study for the detection of total and neutralizing antibodies were validated with the First WHO International Standard Anti-SARS-CoV-2 Immunoglobulin (Cat# 20/136 and #20/268), kindly provided by PATH.  A standard curve was used to plot the neutralization response in the samples as international units (IU) using the WHO International Standard for SARS-CoV-2 antibody (NIBSC code 20/136), which was prepared according to the instructions of the manufacturer [1]. Data were analyzed using a sigmoidal curve model with a logarithm transformation of the concentration, and the final concentration for each sample was the average of the product of the interpolated IU from the standard curve and the sample dilution factor required to reach the OD450 value that falls within the linear range determined for each sample. Samples with undetermined concentration at the lowest dilution tested (1:4) were assigned to the lower limit of quantification (16.4 IU). The Geometric Mean Units (GMU) and titers (GMT) were represented in **Fig. 2** and **Supplementary Fig. 1,** respectively**. Table 2** shows comparisons among the visits.

Conventional virus neutralization tests (cVNT) were performed in Vero E6 cells infected with a SARS-CoV-2 strain obtained by viral isolation in tissue cultures (33782CL-SARS-CoV-2 strain, D614G variant). Neutralization assays were carried out by the reduction of cytopathic effect (CPE) in Vero E6 cells (ATCC CRL-1586). The titer of neutralizing antibodies was defined as the highest serum dilution that neutralized virus infection, at which the CPE was absent as compared with the virus control wells (cells with CPE). Vero E6 cells were seeded in 96-well plates (4×10^4^ cells/well). For neutralization assays, 100 µL of 33782CL-SARS-CoV-2 (at a dose of 100 TCID_50_) were incubated with serial dilutions of heat-inactivated sera samples (dilutions of 1:4, 1:8, 1:16, 1:32, 1:64, 1:128, 1:256, and 1:512) from participants for 1h at 37 °C. Cytopathic effect on Vero E6 cells was analyzed 7 days after infection.

Pseudotyped virus neutralization test (pVNT) was also performed in order to evaluate the neutralizing antibodies against D614G, Gamma and Omicron variant. Antibodies with neutralizing capacities were measured using an HIV-1 backbone expressing firefly luciferase as a reporter gene and pseudotyped with the SARS-CoV-2 spike glycoprotein (HIV-1-SΔ19) from lineage B.1 (D614G), Delta (T19R, del157/158, L452R, T478K, D614G, P681R, D950N) and Omicron (A67V, ∆H69-V70, T95I, Y145D, ∆G142 -V143-Y144, ∆N211, EPE 213-214, G339D, S371L, S373P, S375F, K417N, N440K, G446S, S477N, T478K, E484A, Q493R, G496S, Q498R, N501Y, T547K, D614G, H655Y, N679K, P681H, N764K, N865K, Q954H, N969K, L981F) as previously described *(13).* Briefly, serum samples were initially diluted 1:4 in DMEM, serially diluted 1:3 up to 1:8,748 and then mixed with approximately 4.5 ng of p24 equivalents of HIV-1-SΔ19 in white 96-well plate. Plates were incubated during 1 hour at 37°C and then 100 mL of DMEM containing 1x10^4^ HEK-ACE2 cells was added to each well. Firefly luciferase activity was measured 48h later, using the Luciferase Assay System (Promega) in a Glomax®-96 microplate luminometer (Promega). Estimation of the ID80 was obtained using a 4-parameter nonlinear regression curve fit measured as the percent of neutralization determined by the difference in average relative light units (RLU) between test samples and pseudotyped virus controls. Also, cVNT assays were performed to assess the capacity of the antibodies against SARS-CoV-2 Delta variant in samples from nineteen volunteers of the seventy-seven previously analyzed by sVNT. Data analyses and statistical analyses were carried out using GraphPad Prism v8.

To assess the cellular immune response, ELISPOT and flow cytometry assays were performed using PBMCs from volunteers at different times: the first dose (pre-immune); two weeks after the second dose; four weeks after the second dose; twenty weeks (or five months) after the second dose; four weeks after the booster (third) dose (**Fig. 1B**). Upon thawing, cells were resuspended in fresh media in a 1:10 dilution to remove DMSO remnants from the freezing media. Then, cells were centrifuged, resuspended in fresh media, and counted in an automated cell counter (Logos Biosystems #L40001). Cells were adjusted to 6x10^6^ cells/mL and kept at 37ºC, 5% CO_2_ for 15 min until use in the corresponding assay. ELISPOT plates containing a PVDF membrane were activated with 15 µL of 70% ethanol (Merck), washed three times with sterile 1x PBS, and then coated with human IFN-γ and IL-4 capture antibodies (1:250 and 1:125, respectively, CTL). After 2h of activation at RT, plates were washed two times with PBS and two times with PBS-Tween 20 0.05%. The stimulus included in these assays considers the use of Mega Pools (MPs) of peptides derived from SARS-CoV-2 proteins, previously described *(31)*. Two MPs composed of peptides from the S protein (MP-S) and the remaining proteins of the viral particle (MP-R) were used, as previously described *(31)*. These peptides were determined *in silico* to optimally stimulate CD4^+^ T cells. Also, two MPs composed of peptides from the proteome of SARS-CoV-2 (CD8-A and CD8-B) were used, as previously described *(31)*. These peptides were determined *in silico* to optimally stimulate CD8^+^ T cells. Also, MP derived from the S protein of SARS-CoV-2 WT, SARS-CoV-2 B1.617.2 MP 4326 (Delta variant), and SARS-CoV-2 B1.1.529 MP 4359 (Omicron variant) [2] were used to evaluate T cell activation at four weeks after the booster dose. As positive controls, an independent stimulation was performed with 5 mg/mL of Concanavalin A (ConA) (Sigma Life Science #C5275-5MG) and with an MP of peptides derived from cytomegalovirus proteins (MP-CMV) for the stimulation of both CD4^+^ and CD8^+^ T cells *(31)*. As a vehicle control, DMSO 1% (Merck #317275) was included. A total of 3x10^5^ cells in 50 µL of media were added to each well containing 50 µL of media with the corresponding stimulus. The final concentration of each stimulus per well was 1 µg/mL (except for ConA and DMSO). Positive controls for ELISPOT assays considered 5x10^4^ cells/well instead of 3x10^5^ cells/well. For ELISPOT assays, cells were incubated for 48h at 37ºC, 5% CO_2_. After incubation, plates were washed 1 time with PBS and 3 times with PBS-Tween20. Then, anti-human IFN-γ (FITC) and anti-human IL-2 (Biotin) antibodies (1:1,000 and 1:1,000, respectively) were added and plates were incubated for 2h, RT. Plates were washed 3 more times with PBS-Tween 20 and then FITC-HRP and Streptavidin-AP (1:1,000) were added and plates were incubated for 1h, RT. After incubation, plates were washed 3 more times with PBS-Tween20. Then, plates were treated with the blue (15 min) and red (15 min) developer solution individually following the recommendations of the manufacturer. Plates were washed with tap water after each developer solution and allowed to dry for 24h prior to reading. To evaluate the number of T cells secreting IFN-γ, IL-4 or both, ELISPOT assays were performed with ImmunoSpot^®^ technology (ImmnunoSpot^®^ #hIFNgIL4-1M-10). Spot Forming Cells (SFCs) were counted on an ImmunoSpot^®^ S6 Micro Analyzer.

To characterize the expression of activation-induced markers (AIM) by T cells, flow cytometry assays were performed. 5x10^5^ cells per well were stimulated as described for the ELISPOT assays and after 24 h of incubation with the stimulus, samples were stained. Staining was performed by incubation for 45 min at 4ºC using the reagents listed in Supplementary **Table 1**. Cells were washed twice with 200 µL of PEB buffer, fixed, and then handed to the Flow Cytometry core facility, for their acquisition in an LSRFortessa X-20 flow cytometer.

Cytokine secreted by PBMCs stimulated with MP peptides was performed on a Luminex 200, using a Milliplex MAP Mouse magnetic bead kit (Merck Millipore), following manufacturer instructions. Supernatants of samples stored at -80ºC were thawed at room temperature and diluted 1:2 before analysis. After 2 h incubation with spectrally encoded beads, coated with analyte-specific biotinylated primary antibodies, samples were incubated with streptavidin R-phycoerythrin.

**References**

1. WHO/BS.2020.2403 Establishment of the WHO International Standard and Reference Panel for anti-SARS-CoV-2 antibody. Available at: https://www.who.int/publications/m/item/WHO-BS-2020.2403. Accessed 8 October 2021.

2. Melo-González F, Soto JA, González LA, et al. Recognition of Variants of Concern by Antibodies and T Cells Induced by a SARS-CoV-2 Inactivated Vaccine. Frontiers in Immunology **2021**; 12.
