# Supplementary material for: A Booster Dose of CoronaVac Increases Neutralizing Antibodies and T Cells that Recognize Delta and Omicron Variants of Concern
Source: mBio. 2022 Aug 10;13(4):e01423-22. doi: 10.1128/mbio.01423-22 (PMC9426482; doi:10.1128/mbio.01423-22)
Supplement: TABLE S3 [file mbio.01423-22-s0008.docx]

**Supplementary Table 3: Reagents used in Flow cytometry**

| **Marker** | **Clone** | **Fluorophore** | **Vendor** | **Dilution** |
| --- | --- | --- | --- | --- |
| CD3 | OKT3 | Alexa Fluor 700 | BioLegend | 1:50 |
| CD4 | RPA-T4 | BV605 | BioLegend | 1:50 |
| CD8 | RPA-T8 | BV650 | BioLegend | 1:50 |
| CD14 | M5E2 | V500 | BD | 1:100 |
| CD19 | HIB19 | V500 | BD | 1:100 |
| CD69 | FN50 | PE | BD | 1:10 |
| CD137 | 4-1BB | APC | BioLegend | 1:50 |
| OX40 | BER-ACT35 | PE-Cy7 | BioLegend | 1:50 |
| Fixable Viability Dye | - | BV510 | BD | 1:1000 |

Reagents used in Flow cytometry
